# Expert proposal to characterize cardiac diseases with normal or preserved left ventricular ejection fraction and symptoms of heart failure by comprehensive echocardiography

**DOI:** 10.1007/s00392-022-02041-y

**Published:** 2022-06-04

**Authors:** A. Hagendorff, A. Helfen, R. Brandt, E. Altiok, O. Breithardt, D. Haghi, J. Knierim, D. Lavall, N. Merke, C. Sinning, S. Stöbe, C. Tschöpe, F. Knebel, S. Ewen

**Affiliations:** 1 Department of Cardiology, University of Leipzig, Liebigstraße 20, 04103 Leipzig, Germany; 2 Department of Cardiology, Kath. St. Paulus Gesellschaft, St-Marien-Hospital Lünen, Altstadtstrasse 23, 44534 Lünen, Germany; 3 Department of Cardiology, Kerckhoff Heart Center, Benekestr. 2-8, 61231 Bad Nauheim, Germany; 4 Department of Cardiology, University of Aachen, Pauwelsstrasse 30, 52074 Aachen, Germany; 5 Klinik für Innere Medizin-Kardiologie and Rhythmologie, Agaplesion Diakonie Kliniken Kassel, Herkulesstrasse 34, 34119 Kassel, Germany; 6 Kardiologische Praxisklinik Ludwigshafen-Akademische Lehrpraxis der Universität Mannheim-Ludwig-Guttmann, Strasse 11, 67071 Ludwigshafen, Germany; 7 Department of Cardiothoracic and Vascular Surgery, German Heart Center Berlin, Augustenburger Platz 1, 13353 Berlin, Germany; 8 Paulinenkrankenhaus Berlin, Klinik Für Innere Medizin Und Kardiologie, Dickensweg 25-39, 14055 Berlin, Germany; 9 Department of Cardiology, University of Leipzig, Liebigstrasse 20, 04103 Leipzig, Germany; 10 Department of Cardiology, University Heart and Vascular Center Hamburg, German Centre of Cardiovascular Research (DZHK), Partner Site Hamburg/Kiel/Lübeck, Martinistrasse 52, 20251 Hamburg, Germany; 11 Berlin Institute of Health at Charité (BIH), Universitätsmedizin Berlin, Augustenburger Platz 1, 13353 Berlin, Germany; 12 BIH Center for Regenerative Therapies (BCRT), Augustenburger Platz 1, 13353 Berlin, Germany; 13 German Centre for Cardiovascular Research DZHK, Partner Site Berlin, Augustenburger Platz 1, 13353 Berlin, Germany; 14 Department of Cardiology, Charité University Medicine Berlin, Campus Virchow Klinikum, Augustenburger Platz 1, 13353 Berlin, Germany; 15 Klinik Für Innere Medizin II, Kardiologie, Sana Klinikum Lichtenberg, Fanningerstrasse 32, 10365 Berlin, Germany; 16 Department of Cardiology, University of Berlin, Campus Charité Mitte, Charitéplatz 1, 10117 Berlin, Germany; 17 Zentrale Notaufnahme and Klinik Für Innere Medizin III, Kardiologie, Angiologie Und Internistische Intensivmedizin, Universitätsklinikum Des Saarlandes, Kirrberger Strasse, 66421 Homburg, Germany

**Keywords:** Echocardiography, HFpEF, Ventricular elasticity, Left ventricular hypertrophy, Speckle tracking, Diagnostic workflow

## Abstract

Currently, the term “heart failure with preserved left ventricular ejection fraction (HFpEF)” is based on echocardiographic parameters and clinical symptoms combined with elevated or normal levels of natriuretic peptides. Thus, “HFpEF” as a diagnosis subsumes multiple pathophysiological entities making a uniform management plan for “HFpEF” impossible. Therefore, a more specific characterization of the underlying cardiac pathologies in patients with preserved ejection fraction and symptoms of heart failure is mandatory. The present proposal seeks to offer practical support by a standardized echocardiographic workflow to characterize specific diagnostic entities associated with “HFpEF”. It focuses on morphological and functional cardiac phenotypes characterized by echocardiography in patients with normal or preserved left ventricular ejection fraction (LVEF). The proposal discusses methodological issues to clarify why and when echocardiography is helpful to improve the diagnosis. Thus, the proposal addresses a systematic echocardiographic approach using a feasible algorithm with weighting criteria for interpretation of echocardiographic parameters related to patients with preserved ejection fraction and symptoms of heart failure. The authors consciously do not use the diagnosis “HFpEF” to avoid misunderstandings.

**Graphical abstract:**

Central illustration: Scheme illustrating the characteristic echocardiographic phenotypes and their combinations in patients with “HFpEF” symptoms with respect to the respective cardiac pathology and pathophysiology as well as the underlying typical disease

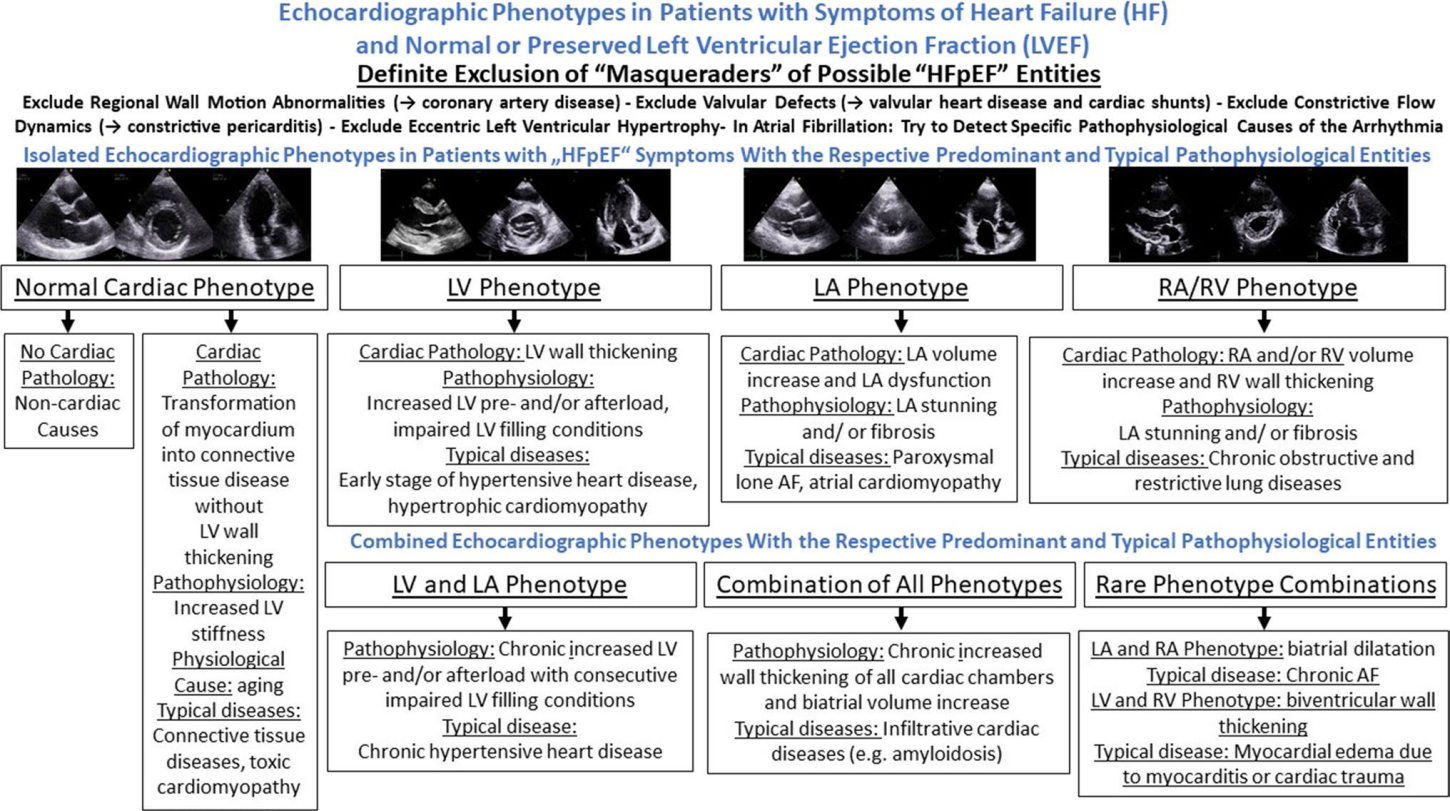

## Introduction

The analysis of left ventricular (LV) function in patients with symptoms of heart failure (HF) is usually performed by transthoracic echocardiography (TTE) as the primary imaging technique in clinical cardiology [1, 2]. Many HF patients with high normal or elevated natriuretic peptides present with a preserved or normal LV ejection fraction (LVEF) above 50% and are currently classified as “heart failure with preserved LVEF” (HFpEF) [3–7]. However, HFpEF should not be considered as a diagnosis, but rather as a syndrome [8]. Multiple pathologic entities may present with the same phenotype “HFpEF”, but with different comorbidities and varying outcomes [9]. Thus, the real diagnostic challenge of TTE in these patients is not to merely measure LVEF, but to characterize the underlying cardiac pathologies and to precisely obtain a specific diagnosis.

“Diastolic HF” was initially defined as “an increased resistance to filling in one or both ventricles, leading to symptoms of pulmonary congestion due to an inappropriate upward shift of the diastolic pressure–volume relation” [3]. Later it was replaced by the term “HFpEF” [5, 6]. The incidence and prevalence of “HFpEF” symptoms approximately constitute half of all HF patients [2, 7]. Recently, diagnostic algorithms have been introduced to characterize patients with preserved LVEF and HF symptoms by the hypernym “HFpEF” as a common *diagnosis* despite different underlying pathologies and treatment options, as well as varied prognosis [7, 9, 10]. The “HFpEF” algorithms are based on clinical complaints, laboratory findings, echocardiographic data and/or invasive hemodynamic measurements [4, 7], which often do not lead to the specific underlying diagnosis [11–18]. If cardiac diagnoses like heart valve disease, significant coronary artery disease, pericardial constriction, et cetera are detected, these specific diagnoses are labeled as “HFpEF masqueraders” and must be excluded [7]. Arrhythmias—most often symptoms due to specific cardiac diagnoses—are also described as “HFpEF” masqueraders, except for atrial fibrillation (AF) which is very frequently observed in patients with “HFpEF” symptoms [19, 20]. To be fair, the exclusion of “HFpEF masqueraders” at best results in the diagnosis “HFpEF” with unknown origin. However, there are several other cardiologic diagnoses like myocarditis, myocardial infarction with non-obstructive coronary artery disease, restrictive and infiltrative cardiomyopathies contributing to the dilemma of multiple components of etiological variety in patients with “HFpEF” symptoms [21–26]. Furthermore, non-cardiac diseases—especially pulmonary diseases, anemia, diabetes, systemic infections, consuming neoplasms, obesity, and frailty—complicate the multifactorial hodgepodge of “HFpEF” as a diagnosis [27–32].

**Fig. 1:**
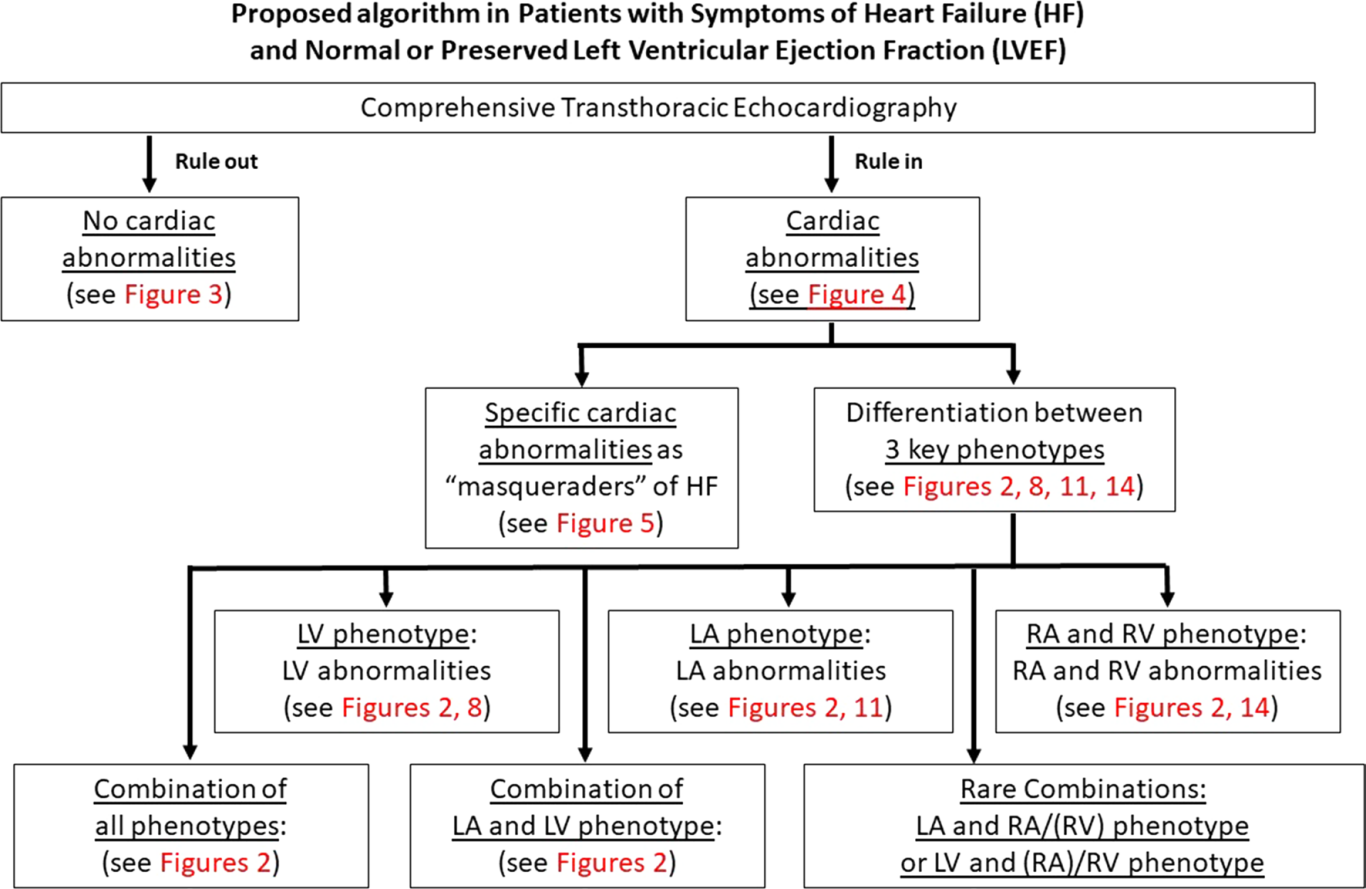
Scheme illustrating the echocardiographic workflow to characterize patients with “HFpEF” symptoms due to cardiac phenotypes

Thus, the present expert proposal focuses on a diagnostic TTE workflow to overcome diagnostic challenges and to increase options for the detection of specific cardiac entities in patients with HF symptoms and preserved LVEF (Fig. 1). With respect to conventional and modern echocardiographic tools the authors try to highlight, why, when, and how to implement special echocardiographic features into clinical practice. In addition, the proposal underlines new aspects, which are presumably worth to be included into the workflow.

**Fig. 2:**
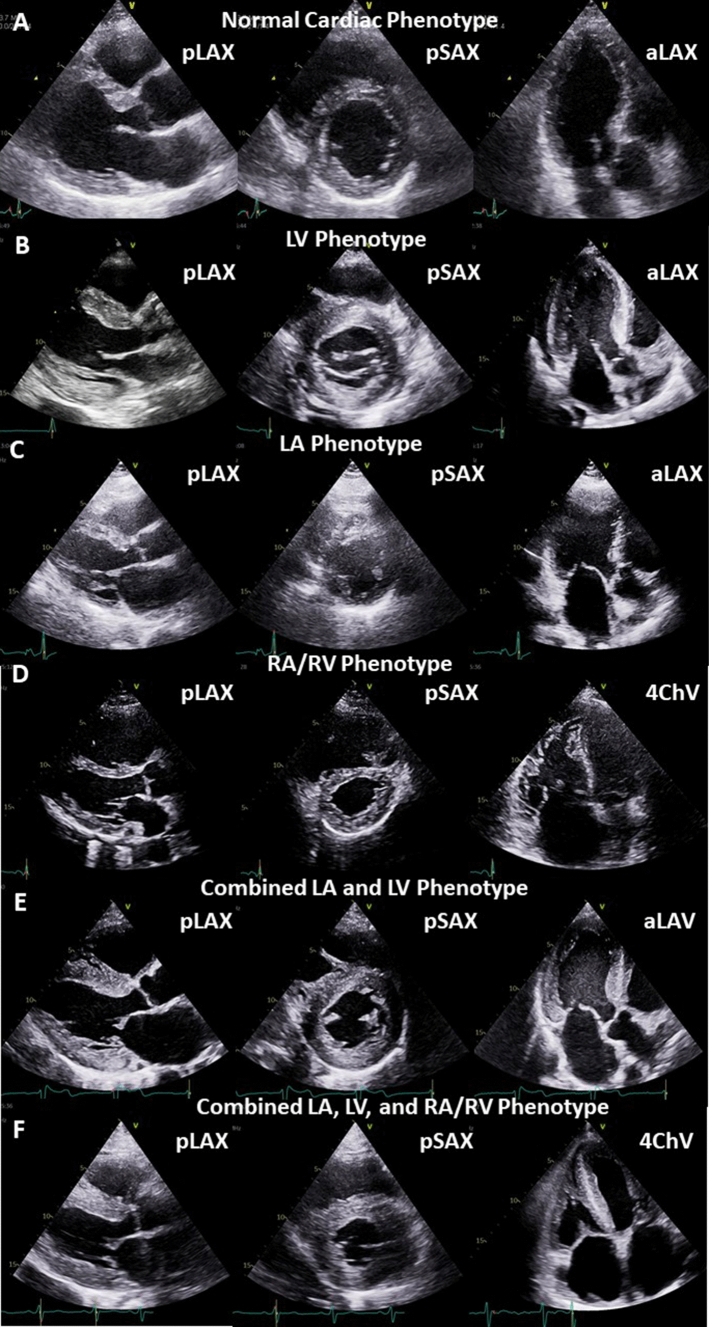
Illustration of cardiac phenotypes in patients with “HFpEF” symptoms by echocardiography. The rows illustrate representative parasternal long and short axis views (pLAX, pSAX) and apical long axis views (aLAX) or apical 4-chamber views (4ChV) of the respective phenotypes. In A the normal cardiac phenotype is shown, in B the isolated LV phenotype (in compensated hypertensive heart disease—short history), in C the isolated LA phenotype (in paroxysmal lone atrial fibrillation), in D the isolated RA/RV phenotype (in chronic pulmonary hypertension due to repetitive pulmonary thromboembolism), in E the combined LA and LV phenotype (in compensated hypertensive heart disease—long history), and in F the combined LA, LV, and LA/RV phenotype (in amyloidosis)

TTE guiding to diagnose specific cardiac pathologies commonly reflects the initial contact of patients undergoing diagnostic cardiological procedures prior to laboratory findings, prior to invasive measurements, and sometimes even prior to interpretation of the electrocardiogram. Thus, the focus of a detailed morphological and functional characterization of cardiac entities by TTE plays a key role of cardiac diagnostics. With respect to morphological and functional alterations of cardiac chambers, the following cardiac phenotypes are outlined (Fig. 2):

1. The normal cardiac phenotype which can be associated with increased LV stiffness in patients with HFpEF symptoms,
2. The LV (= left ventricular) phenotype which is associated with morphological or functional abnormalities of the LV wall,
3. The LA (left atrial) phenotype which is solely associated with an increase of LA size and consecutive impairment of LA function,
4. The RA (= right atrial) and RV (= right ventricular) phenotype which is solely associated with morphological and functional abnormalities of right cardiac chambers,
5. The combination of LA and LV phenotype,
6. The combination of all three phenotypes and
7. Rare combinations of LA and RA/(RV) phenotype or LV and (RA)/RV phenotype.

The introduction of isolated echocardiographic phenotypes is proposed with respect to diagnostic systematics to focus on the predominant morphological or functional cardiac abnormality. The classification of patients with “HFpEF” symptoms according to these morphological and functional cardiac entities is still challenging due to the complex interactions of pathophysiological factors on the heart. Hence, majority of patients with “HFpEF” symptoms have combinations of these echocardiographic phenotypes.

The characteristic echocardiographic phenotypes and their respective combinations are depicted with respect to the representative diseases in the central illustration (central illustration). In addition, the cardiac pathology and pathophysiology as well as typical representative diseases are highlighted to demonstrate the diagnostic pathway depending on echocardiographic morphological classification.

## Targets of echocardiography in patients with heart failure and preserved ejection fraction

A proper LVEF assessment is the first diagnostic step to identify HF patients with preserved LVEF. The recommended quantitative LVEF assessment is mostly performed by biplane planimetry using the modified Simpson method [33]. The two-dimensional approach from the apical four- and two-chamber view often results in underestimation of LV cavity size by apical foreshortening. Nowadays, standardization of apical LV views can be ensured using triplane or multidimensional TTE [33–35]. Thus, these modern modalities should be generally preferred, especially in the presence of regional wall motion abnormalities and pathological LV geometry. According to recent recommendations the parameter LVEF is still predominantly used to differentiate between normal, preserved, mildly, moderately, or severely reduced LV function [2]. Total LV stroke volume (LVSV_tot_) measured by 2D planimetry or 3D volumetry reflects effective (systemic) LV stroke volume (LVSV_eff_) if patients with significant aortic and/or mitral regurgitation are excluded. Stroke volume can also be estimated using Doppler echocardiography in the LV and right ventricular (RV) outflow tract (LVOT, RVOT). LVSV_tot_ and LVSV_eff_ should be in the same range if no relevant valvular heart disease is present and can be counterchecked to verify hemodynamic plausibility of the measurements [33–35]. In case of limited acoustic windows, optimization of LV endocardial border detection by contrast echocardiography should be considered for LV volume assessment [33, 35, 36].

**Fig. 3:**
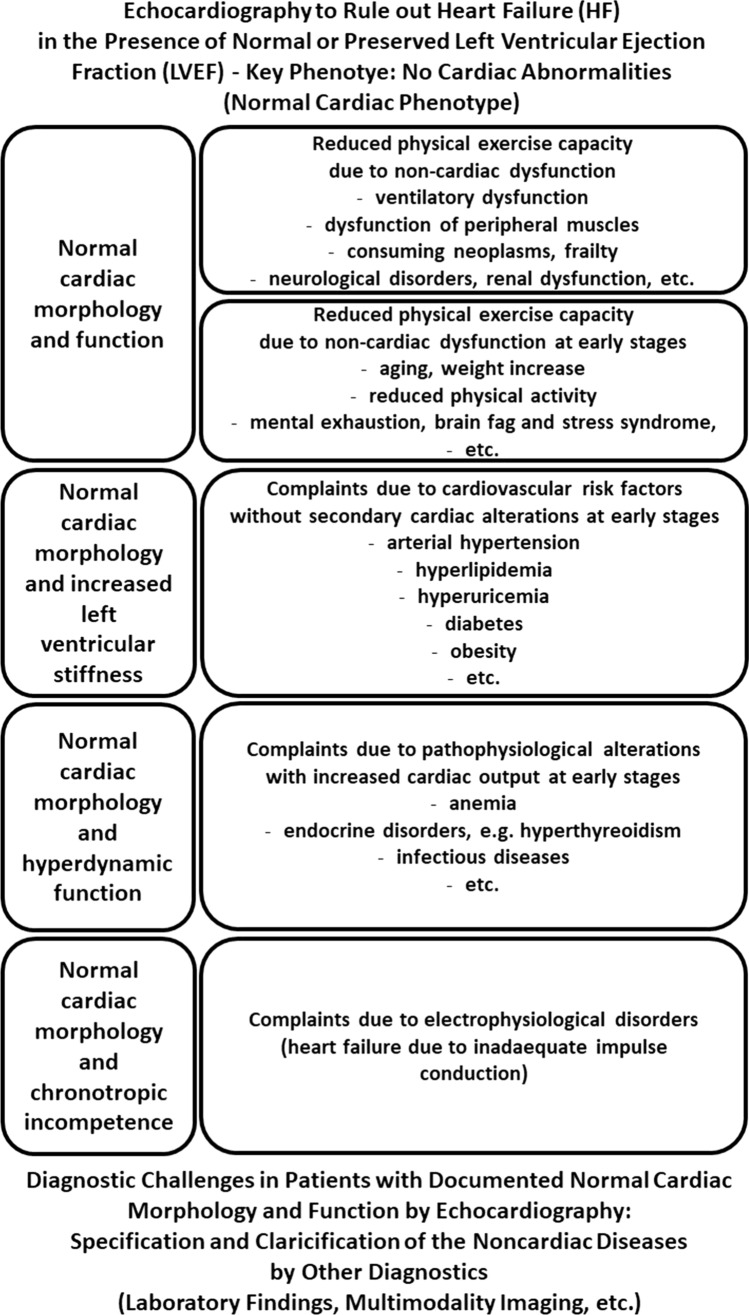
Scheme of echocardiographic workflow describing the tool to rule out cardiac manifestations as the primary cause of “HFpEF” symptoms

**Fig. 4:**
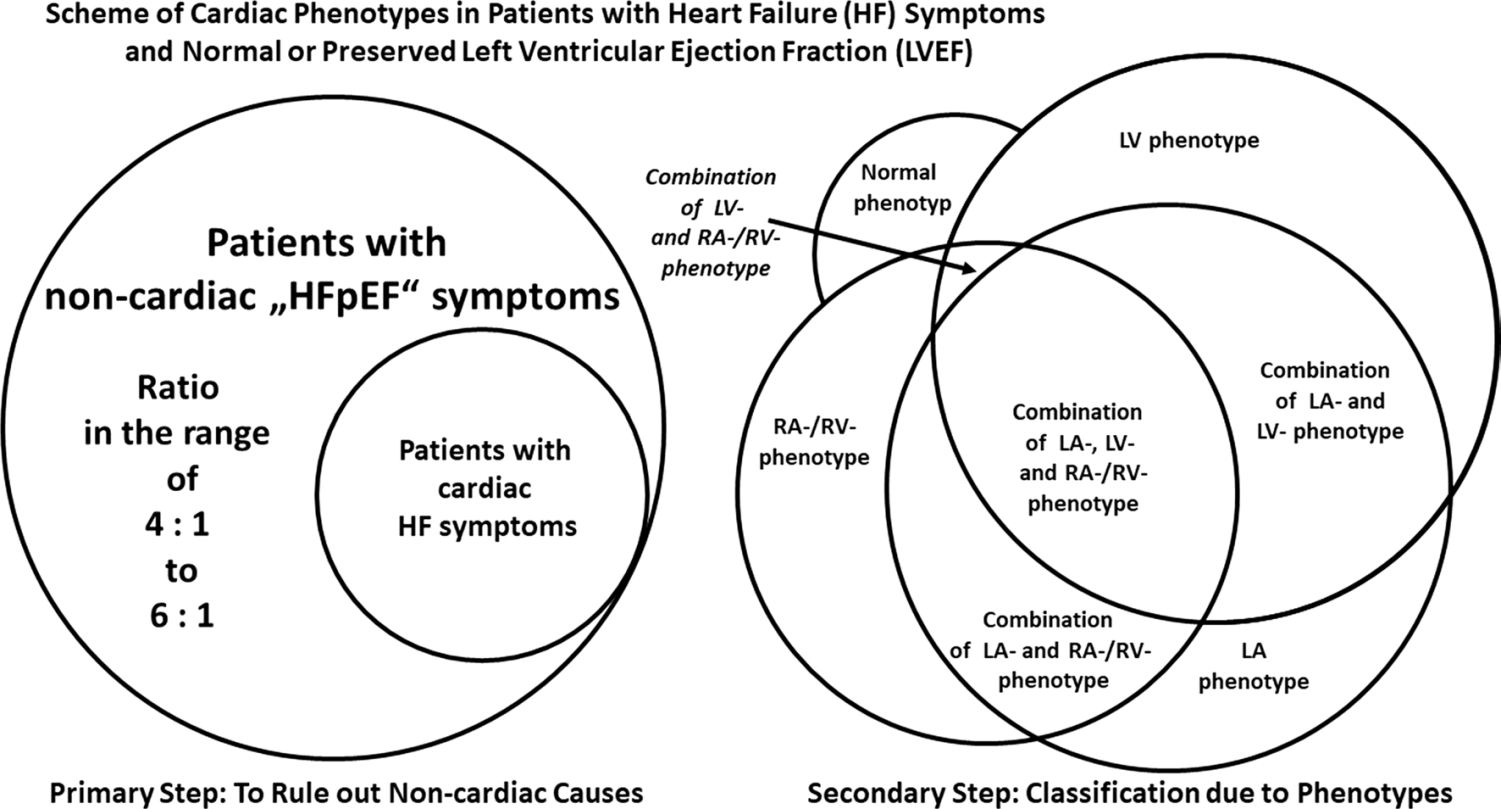
Scheme of the ratio between patients with noncardiac and cardiac HF symptoms (left)—scheme of the interactions/combinations between cardiac phenotypes in patients with “HFpEF” symptoms

The first integral component of HF diagnostics by TTE in patients with “HFpEF” symptoms (predominantly dyspnea or disability) is to rule-out or to rule-in HF as the reason for clinical symptoms [2]. Cardiac morphology and function should be classified in relation to the cardiac phenotypes and their combinations to structure the echocardiographic workflow cardiac in patients with “HFpEF” symptoms (Fig. 2). If no abnormalities are detected by conventional echocardiography, a “normal cardiac phenotype” refers to a non-cardiac entity of the “HFpEF” symptoms (Figs. 3, 4). The pathophysiological reason for “HFpEF” symptoms in “normal cardiac phenotype” patients can be explained by the mismatch between oxygen supply and oxygen demand due to high output stages, chronotropic incompetence, and/or subclinical stages of unknown cardiac diseases. Non-cardiac disease-related “HFpEF” symptoms might be caused by a high cardiac output (CO) due anemia, hyperthyroidism, liver cirrhosis, infectious diseases, and/or obesity due to different pathophysiologies. Notably, the measurement of CO is mandatorily in patients with dyspnea and normal cardiac phenotype if LV hypercontractility and brady- or tachycardia is observed. The problem of the echocardiographic “normal cardiac phenotype” is to miss early echocardiographic signs related to LV and left atrial (LA) wall thickness, stiffness, and function. Thus, this scenario describes the diagnostic challenge to detect early or preclinical disease stages with subtle echocardiographic signs of pathological cardiac phenotypes.

The proposed pathological echocardiographic phenotypes can be subdivided into a LV phenotype, a LA phenotype, and a combined right atrial (RA) and RV phenotype (Figs. 1, 2). The diagnostic aspects of these pathological phenotypes in patients with “HFpEF” symptoms are described in detail in the following paragraphs. They address the second step of TTE after the rule-in of HF as the cause of the symptoms. We believe that this proposed classification of echocardiographic phenotypes will help to improve the identification of a specific underlying diagnosis for HF. The third task of echocardiography in HF patients is to monitor specific treatment effects during follow up and to estimate individual patient’s prognosis.

## The echocardiographic characterization of patient with “HFpEF” symptoms using recent “HFpEF” algorithms: what is useful?

“New” paradigms—e. g. the identification of a systemic proinflammatory state induced by comorbidities as the cause of myocardial structural and functional alterations—have been proposed to explain the genesis of “HFpEF” symptoms [10, 11] and new scores have been introduced to overcome the challenges of diagnostics in patients with “HFpEF” symptoms [13–18].

After LVEF determination the second pivotal finding by TTE in patients with “HFpEF” symptoms is the increased ratio of E/E´ (E = early mitral flow velocity, E´ = early tissue Doppler lengthening velocity of the myocardium near to the mitral annulus) [4, 7]. Interestingly, increased NT-proBNP- and BNP levels (NT-proBNP = N-terminal-pro brain natriuretic peptide, BNP = brain natriuretic peptide) are not necessarily measured in patients characterized by these echocardiographic criteria. Invasive hemodynamic measurements of PCWP, LVEDP (PCWP = pulmonary capillary wedge pressure, LVEDP = left ventricular end diastolic pressure), the time constant of ventricular relaxation τ and LV chamber stiffness are proposed as an alternative to prove cardiac cause of “HFpEF” symptoms, but often not performed [2, 4, 7]. Thus, echocardiography should implement additional parameters beside the documentation of E´, E/E´, and systolic pulmonary artery pressure (sPAP) to characterize diastolic dysfunction (DD) in patients with “HFpEF” symptoms [37–42].

**Fig. 5:**
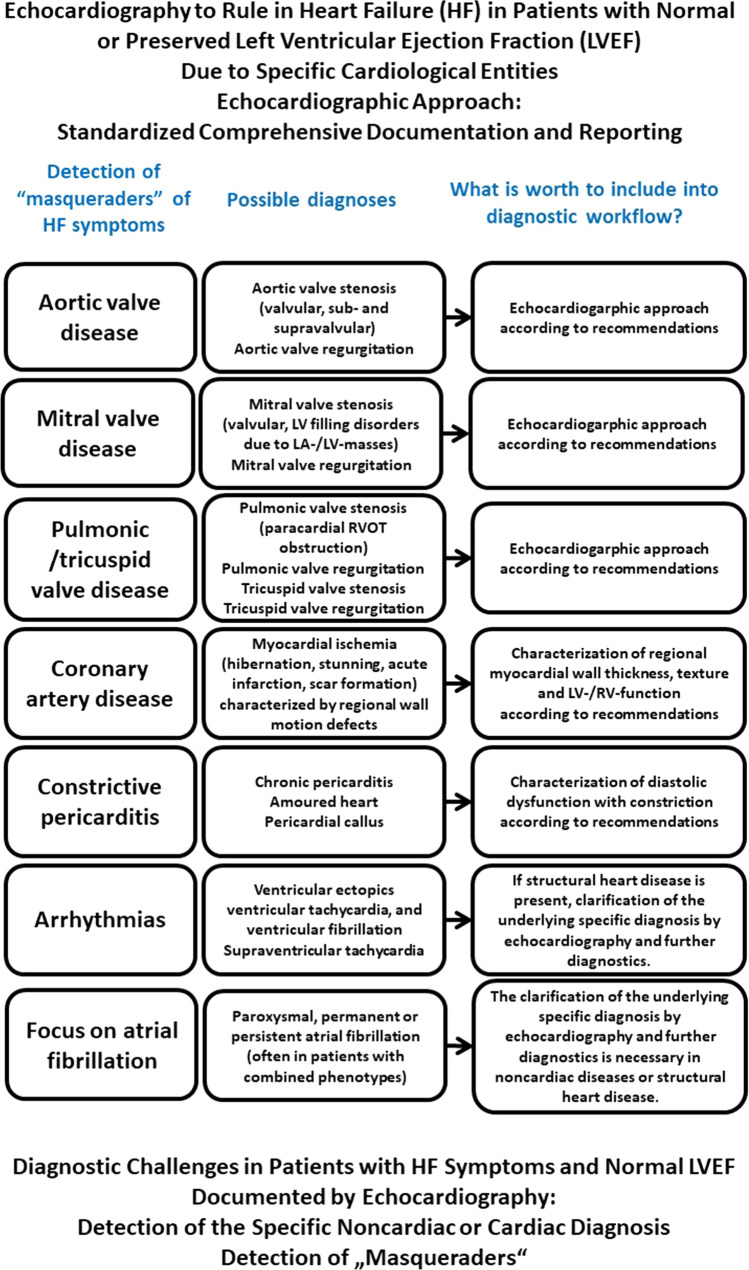
Scheme of the echocardiographic workflow in the presence of “HFpEF masqueraders”. “HFpEF masqueraders” are specific detected diagnoses in patients with “HFpEF” symptoms

According to recent recommendations, the second target in patients with “HFpEF” symptoms is the detection of the “masqueraders” (Fig. 5), as described above [4, 7]. The list of “masqueraders” might be complemented by all specific diagnoses which can be detected by a comprehensive echocardiography to minimize the usage of “HFpEF” with unknown origin as a final diagnosis. In addition, the underlying diagnoses in the presence of arrhythmias—including AF—should also be clarified in patients with HF symptoms and preserved LVEF.

After ruling out patients with non-cardiac causes and excluding those with a known specific cardiac diagnosis the remaining cohort of patients is composed of patients with hypertensive heart diseases, infiltrative/storage diseases, and unknown specific diagnoses. Because clinical and echocardiographic abnormalities can be observed in subgroups of this patient cohort several phenotypes have also been introduced in the literature, i. e. phenotypes associated with LA abnormalities, with pulmonary hypertension and pulmonary disease in the presence of RV dysfunction, with obesity combined with cardiac abnormalities, and with the ischemia/microvascular dysfunction [39, 43–46]. However, several further entities like aging, myocardial LA and LV stiffness, systemic hypertension, high output stages, and lone AF can be added illustrating the diversity within this remaining cohort of patients with HF symptoms and preserved LVEF.

Thus, we like to propose the following phenotypes, solely described by echocardiographic findings: the LV phenotype, the LA phenotype, and the combined RA and RV phenotype (Fig. 2).

## The LV phenotype: abnormalities of LV volumes, LV function, LV geometry, and LV mass

### Conventional echocardiographic analyses of LV wall thickness, LV volumes and LVEF

This phenotype addresses LV abnormalities characterized by TTE. Despite LV planimetry or LV volumetry is possible in native 2D- and 3D echocardiography with adequate image quality endocardial contour delineation can often be improved by LV opacification using contrast to avoid underestimation of LV volumes [33–36, 47]. Modern modalities of triplane and 3D echocardiography are strongly recommended to avoid foreshortening which leads to smaller LV volumes and underestimation of LV function. Triplane and 3D echocardiography modalities are particularly useful in patients with regional wall motion abnormalities and pathological LV geometry.

The conventional echocardiographic M-Mode and 2D parameters characterizing LV dimensions, LV wall thickness, relative wall thickness (RWT), LV mass are most useful and reproducible in normal LV geometry and during normal cardiac conditions [33, 48]. If LV abnormalities are detectable and if the acoustic window is appropriate, LV wall thickness, LV volumes, LV wall stress and LV remodeling index can also be assessed by 3D echocardiography to distinguish between normal conditions, LV remodeling, concentric and eccentric LV hypertrophy [33–36, 47]. Standardization of M-Mode measurements can be achieved by anatomical M-Mode in biplane image acquisition to provide appropriate sectional planes through the LV center rather than LV secants, and LV measurements perpendicular to the LV long axis at the level of the mitral valve (MV) leaflet tips. A high spatial resolution using the best acoustic window usually enables the exclusion of right and left ventricular trabecula for appropriate delineation of LV wall diameters.

### Strain analyses of GLS and myocardial work

Specific echocardiographic modalities should be used to demarcate subclinical and/or subtle findings as a screening tool in this predefined patient cohort. Global longitudinal strain (GLS) is nowadays an established parameter to characterize LV function as well as prognosis [49, 50]. Global circumferential and global radial strain (GCS, GRS) are also detectable by speckle tracking echocardiography, but are still not generally used. Regional myocardial dysfunction due to local ischemia, scar, hypocontractility or dyssynchrony can be visualized and quantified by the segmental 2D strain curves, which are often showing disease specific deformation patterns. When compared to the conventional LVEF assessment, the quantification of GLS is much more sensitive for the detection of subtle impairment of LV function like in early stages of infiltrative/storage diseases—especially in patients with amyloidosis—as many pathologies tend to primarily affect the longitudinal LV deformation [51, 52]. The conventional evaluation of LV longitudinal deformation by M-mode measurement of mitral annular plane systolic excursion (MAPSE) is a valid surrogate marker for GLS, but without regional/segmental information, thus GLS by 2DS should be preferred [53]. The different strain distribution patterns are associated with different underlying pathologies. The presence of subclinical fibrosis, e.g. in hypertensive heart disease, toxic, immune, inflammatory, infiltrative, metabolic, genetic and endomyocardial myocardial pathologies, typically affects more the basal and mid LV segments, [7, 49, 54], while the “apical sparing” pattern with a preserved regional strain only in the apical segments is typically seen in patients with cardiac amyloidosis. Predominant alterations of regional circumferential and radial strain are observed in patients with myocardial edema due to myocarditis or cardiotoxic agents [55–57]. Thus, the analysis of the systolic LV contraction patterns should include the analysis of all interacting compounds of LV deformation.

LV function must be considered in relation to LV contraction, which is strongly dependent on pre- and afterload. Myocardial work can be non-invasively analyzed by pressure–strain loops to (partially) overcome the load-dependent limitations of LVEF and GLS. The parameter global work index (GWI), global constructive work (GCW), global wasted work (GWW), and global work efficiency (GWE) can be determined, if blood pressure is added to the strain analysis measured simultaneous to the TTE [58, 59]. The pressure–strain loop area reflects myocardial metabolic demand and oxygen consumption providing information about myocardial energetics [58, 59]. In patients with “HFpEF” symptoms and normal cardiac phenotype or LV phenotype differentiation between hypertensive and coronary heart disease is facilitated because myocardial work incorporates deformation and loading conditions into the analysis [58–61]. Thus, strain patterns with regional decreased longitudinal strain correspond to myocardial work patterns, whereas normalization of myocardial work patterns can be observed in hypertensive heart disease.

### Analysis of LV stiffness, LV elastance, and arterial elastance

**Fig. 6:**
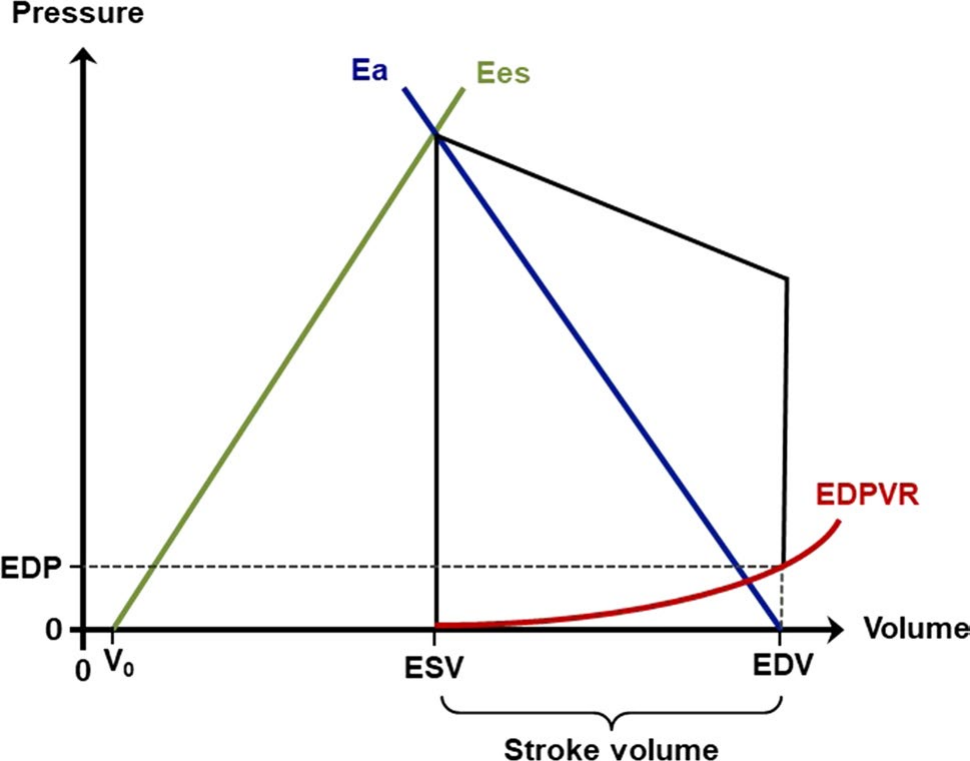
Scheme of a non-invasive LV pressure volume loop to explain the calculations of the end-systolic pressure volume relationship (ESPVR) by its slope which defines the end-systolic elastance (Ees), and by the slope between the end-systolic pressure–volume point and the end-diastolic volume at a LV pressure of 0 mmHg, which defines arterial elastance (Ea) representing LV afterload. EDP, end-diastolic pressure; EDPVR, end-diastolic pressure volume relationship; EDV, end-diastolic volume; ESV, end-systolic volume; V_0_, x-axis intercept of Ees

Doppler and tissue Doppler echocardiographic parameters have been evaluated in comparison to invasive measurements to estimate diastolic function and LV filling pressures favoring the echocardiographic parameter of the lateral E´ [62]. However, the estimation of LV filing pressures solely by echocardiographic parameters has several limitations, especially in patients with regional LV wall motion abnormalities [63, 64]. In patients with “HFpEF” symptoms, a further approach to detect subtle alterations of the LV myocardium might be the assessment of diastolic stiffness, myocardial relaxation, and resulting LV contractility by the analysis of LV end-diastolic volume (LVEDV) in relation to LVEDP [65–67]. The invasive LV pressure–volume relationship is recognized as the gold standard to analyze these parameters. It requires the generation of several pressure–volume loops in different preload states. The approximately linear relationship of their end-systolic pressure–volume points represents the end-systolic pressure volume relationship (ESPVR). The slope of the ESPVR defines the end-systolic elastance (Ees), which serves as a measure of myocardial contractility (Fig. 6). Compared to LVEF or GLS, Ees exhibits a significant correlation to increased global myocardial afterload characterized by arterial elastance (Ea). The increased steepness and left-shift of ESPVR with increasing inotropy represents the myocardial homeometric autoregulation known as Anrep effect [68–70]. Graphically, Ea is reflected by the negative slope between the end-systolic pressure–volume point and the end-diastolic volume at a LV pressure of 0 mmHg (Fig. 6). Ea incorporates arterial compliance and vascular resistance but is also affected by heart rate. In healthy individuals, Ea and Ees are well connected to provide optimal mechanical ventricular-arterial coupling with an Ea/Ees ratio between 0.5 and 1.0 [71]. The indirect measurement of Ees has been developed using a single-beat non-invasive approach to calculate Ees and Ea from pressure and volume measurements during one cardiac cycle [72, 73]. Specifically, the pressure–volume loop is computed from LVEDV and LV end-systolic volume (LVESV), measured by TTE, and blood pressure, measured non-invasively. For Ees calculation, systolic time intervals, defined by pre-ejection period and systolic ejection time, are determined from pulsed-waved Doppler in the LVOT with simultaneous ECG recording. Non-invasive single beat pressure–volume analysis has been applied to study the pathophysiology, prognosis [65–67] as well as the effect of therapeutic HF interventions [74–76]. However, due to the complexity of non-invasive Ea and Ees determination this method is not commonly used and needs further evaluation.

**Fig. 7:**
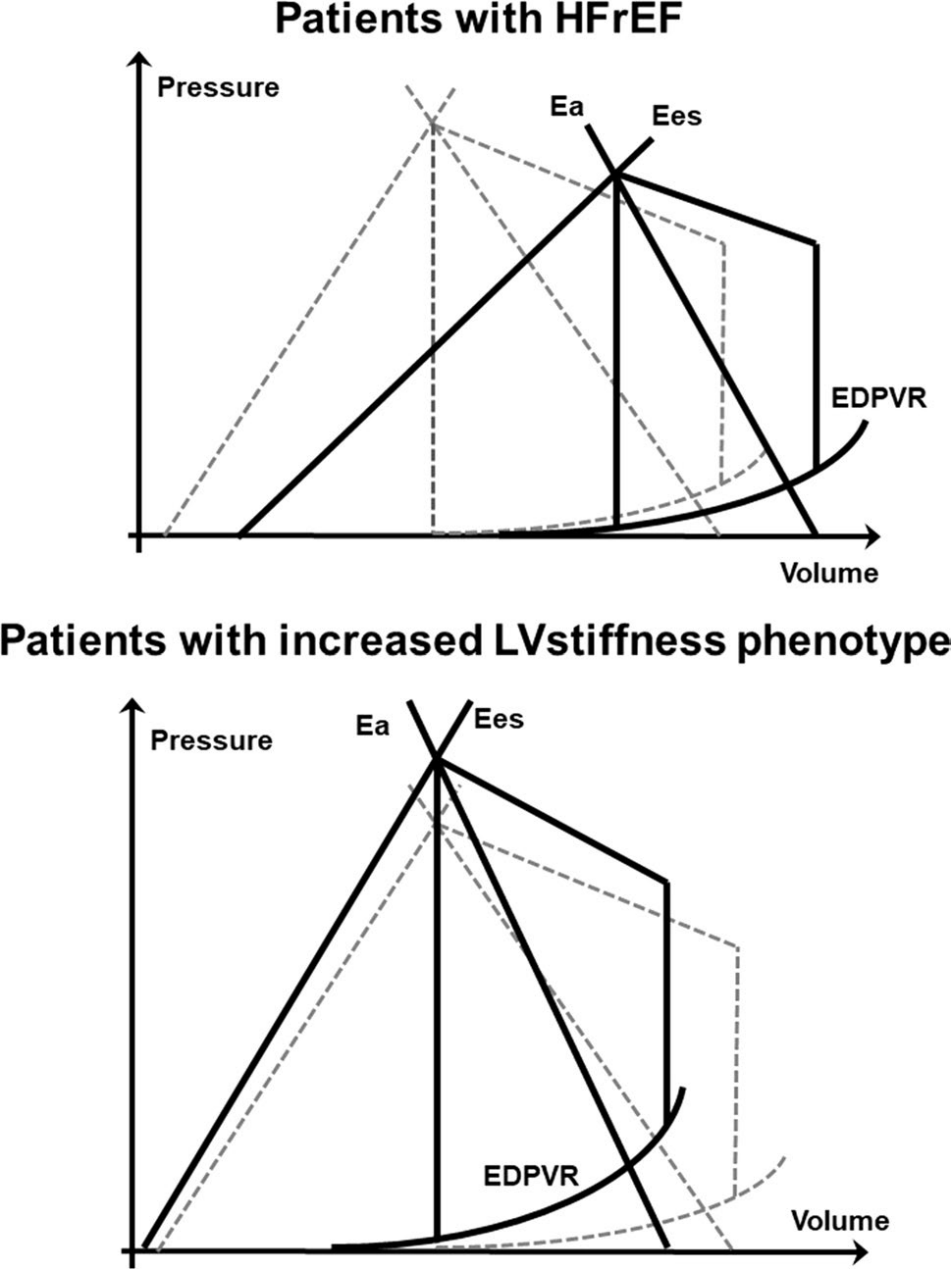
Characteristic pressure–volume loops in patients with heart failure with reduced (HFpEF; top)and preserved (HFpEF; bottom) ejection fraction. In HFrEF patients, end-systolic elastance (Ees) is reduced and LV volumes are increased, thus LV pressure–volume loop is right-ward shifted. In patients with “HFpEF” symptoms and increased LV stiffness, LV end-diastolic volume is reduced due to concentric remodeling, which leads to a leftward shifted end-diastolic pressure volume relationship (EDPVR), i. e. increased LV filling. Ea is frequently elevated, Ees normal or increased. The pressure–volume curves of a non-failing heart with its EDPVR, Ees and Ea is drawn with dotted lines. The schemes explain the increase of LV stroke volume (LVSV_tot_) in HFrEF patients and the minor changes of LVSV_tot_ in patients with “HFpEF” symptoms with afterload reduction

In HF patients with reduced ejection fraction (HFrEF), Ees is reduced due to impaired myocardial contraction, while Ea might be elevated despite normal blood pressure. Hence, Ea/Ees increases in HFrEF patients, indicating ineffective arterial-ventricular coupling (Fig. 7) [66, 71]. An important hemodynamic characteristic in patients with normal LV size and normal or increased LV wall thickness and “HFpEF” symptoms (normal cardiac phenotype and LV phenotype) and in HFrEF patients is the response in LV ejection to changes in afterload. In HFrEF patients, LV stroke volume is more sensitive for afterload changes than in the non-failing heart or in this defined phenotype of patients with “HFpEF” symptoms—which can be labelled as an increased LV stiffness phenotype—because the slope of ESPVR (Ees) is flat (Fig. 7). Thus, afterload reduction, for instance using vasodilators, reduces Ea, which is frequently elevated in HFrEF patients, but lowers blood pressure only slightly. Thus, LVSV_tot_ increases. In increased LV stiffness phenotype Ees is high or normal because LV contractility is preserved (Fig. 7). Vasodilators reduce Ea and blood pressure significantly but LVSV_tot_ would remain relatively constant [77]. Both patients with hypertensive heart disease and with “HFpEF” symptoms show increased Ea values, and Ees increases to compensate for elevated Ea. Thus, ventricular-arterial coupling remains in the normal range. When HF symptoms occur, patients with “HFpEF” symptoms are characterized by increased myocardial stiffness and reduced myocardial contractility—both impact Ees—keeping Ees at a high level. Hence, Ees does not specifically characterize patients with “HFpEF” symptoms nor does it predict outcome [65].

The single-beat method to assess Ees and Ea allows also to calculate the end-diastolic pressure–volume relationship (EDPVR) (Fig. 6) [78]. EDPVR is built from echocardiographic measurement of LVEDVs and the calculation of LVEDP, derived from Doppler and tissue Doppler (E/E’) parameters [78]. Thus, the increased LV stiffness phenotype is also characterized by increased diastolic stiffness and impaired myocardial relaxation, leading to reduced LVEDVs at similar LVEDPs compared to patients with hypertension without HF symptoms and healthy controls [79]. In consequence, the EDPVR curve is leftward-shifted in patients with “HFpEF” symptoms; in contrast, EDPVR is rightward-shifted in HFrEF patients compared to non-heart failure patients (Fig. 7). Both alterations of EDPVR have prognostic implications [67, 80].

In summary, non-invasive single-beat analysis of the LV pressure–volume relationship is based on proper measurements by TTE in combination with simultaneous non-invasive blood pressure measurements [72, 73, 78]. It allows to evaluate LV systolic and diastolic function, LV afterload, and ventricular–arterial coupling. The pressure–volume analysis of the LV provides important insights into the specific pathophysiology and hemodynamics in patients with “HFpEF” symptoms defining a LV stiffness sub-phenotype. The analysis of LV stiffness might be helpful to detect subclinical stages of cardiac diseases, but still needs validation in further clinical studies.

Table 1 summarizes the echocardiographic parameters, which are important to differentiate between the normal cardiac phenotype and the LV phenotype. The parameters are listed including normal ranges and cut offs [33, 72, 81–88], methodological aspects of TTE determination, their importance to be determined, and their reasons, why it is worth to determine the respective parameter in clinical routine. The intention of the tables is to list these important echocardiographic parameters for the sake of completeness as an easy reference for the user. Thus, the authors do not mandatory propose to measure of all these parameters.

**Table 1:** Echocardiographic parameters characterizing patients with heart failure (HF) symptoms and normal or preserved left ventricular ejection fraction (LVEF) in normal cardiac phenotype and the left ventricular (LV) phenotype

Echocardiographic parameter	normal ranges—cut offs	Methodological aspects	Mandatory to determine (methods)	Why worth to do in routine
LV parameters				
** LVEDD—left ventricular end diastolic diameter (mm)**	**♂ 36–56** **cut off ≤ 58** **♀ 35–51** **cut off ≤ 52** [84, 85]	**2D or anatomical M-Mode: avoid oblique measurements using conventional M-Mode, define the LV level at MV leaflet tips**	**Yes**	**To estimate LV size**
** LVESD—left ventricular end sytolic diameter (mm)**	**♂ 21–41** **cut off ≤ 40** **♀ 21–37** **cut off ≤ 35** [84, 85]	**2D or anatomical M-Mode: see above**	**Yes**	**To estimate LV size**
** LVEDV—left ventricular end diastolic volume (ml)**	**♂ 72–204** **cut off ≤ 204** **♀ 61–144** **cut off ≤ 144** [81]	**2D planimetry (biplane or triplane) or 3D volumetry: if regional wall motion defects are present use triplane or 3D volumetry, avoid foreshortening**	**Yes**	**To estimate LVEDV—especially in LV remodeling**
** LVESV—left ventricular end systolic volume (ml)**	**♂ 28–83** **cut off ≤ 83** **♀ 21–61** **cut off ≤ 61** [81]	**2D planimetry (biplane or triplane) or 3D volumetry: see above**	**Yes**	**To estimate LVEF and total LVSV**
** LVSV**_**tot**_** (total left ventricular stroke volume) = LVSV**_**eff**_** (effective left ventricular stroke volume)**	**♂ 41–115** **cut off > 41** **♀ 36–88** **cut off > 36** [81]	**2D planimetry (biplane or triplane) or 3D volumetry: see above**	**Yes**	**To estimate LVEF and total LVSV, which corresponds to effective LVSV, if AV and MV are normal**
D_LVOT_ (mm)—diameter of the left ventricular outflow tract (LVOT)	♂ 18–26 ♀ 17–23 [85]	2D imaging—biplane imaging preferred with respect to verifiable standardization	Yes—under certain conditions	To estimate LVSV_eff_ and cardiac output (CO)
Cardiac output (CO) determined by LVSV_eff_ using pulsed waved (pw) Doppler x heart rate (ml/l)—LVSV_eff_ = 0.785 × D_LVOT_^2^ × VTI_LVOT_ (velocity time integral determined in the LVOT)	♂ 3.5–8.2 ♀ 3.3–7.3 [88]	Proper positioning of the pw sample volume in relation to the D_LVOT_ is mandatory—angulation of the cursor parallel to the blood stream is mandatory	Yes—under certain conditions	To estimate LVSV_eff_ and CO To compare LVSV_tot_ and LVSV_eff_
** LVEF—left ventricular ejection fraction (%)**	**♂ 49–67** **cut off ≥ 55** **♀ 51–79** **cut off ≥ 52** [1, 7]	**2D planimetry (biplane or triplane) or 3D volumetry: see above**	**Yes**	**To estimate normal or preserved LVEF**
LVEDV/BSA (body surface area) (ml/m^2^)	♂ 41–97 cut off < 75 ♀ 39–82 cut off < 62 [81, 84]	Determination of BSA by body height and body weight, 2D planimetry (biplane or triplane) or 3D volumetry: see above	Yes—under certain conditions	To adjust LV volumes in extreme conditions
LVESV/BSA (ml/m^2^)	♂ 16–42 cut off < 32 ♀ 14–35 cut off < 25 [81, 84]	Determination of BSA by body height and body weight, 2D planimetry (biplane or triplane) or 3D volumetry: see above	Yes—under certain conditions	To adjust LV volumes in extreme conditions
** EDLVs—enddiastolic septal LV wall thickness (mm)**	**♂6–12** **cut off < 12** **♀ 5–11** **cut off < 11** [85]	**2D or anatomical M-Mode: avoid oblique measurements, avoid inclusion of RV and LV trabecula, define the LV level at MV leaflet tips**	**Yes**	**To detect or exclude LV hypertrophy**
**EDLVp—enddiastolic posterior LV wall thickness (mm)**	**♂ 6–13** **cut off < 13** **♀ 6–12** **cut off < 12** [85]	**2D or anatomical M-Mode: see above**	**Yes**	**To detect or exclude LV hypertrophy**
** RWT (relative wall thickness) = 2 EDLVp/LVEDD -**	**♂ 0.24 – 0.42** **cut off < 0.42** **♀ 0.22 – 0.42** **cut off < 0.42** [33]	**2D or anatomical M-Mode: see above**	**Yes**	**To detect remodeling and to distinguish between concentric and eccentric LVH**
LV mass (g)—linear method (Cube formula)—3D analysis (3D based formula) (formula see [8])	♂ 88–224 cut off ≤ 224 ♀ 67–162 cut off ≤ 162 [33, 84, 85]	2D or anatomical M-Mode or 3D volumetry: if image quality is good, 3D assessment is preferred	Yes—under certain conditions	To determine severity of LVH
**LV mass/BSA (g/m**^**2**^**)—linear method (Cube formula)—3D analysis (3D based formula) (formula see **[8]**)**	**♂ 49–115** **cut off ≤ 102** **♀ 43–95** **cut off ≤ 88** [33, 84]	**Determination of BSA by body height and body weight, 3D volumetry: if image quality is good, 3D assessment is preferred**	**Yes**	**To determine severity of LVH**
LVRI—left ventricular remodeling index (g/ml) = LV mass/LVEDV (formula see [4])	0.79–1.27 cut off < 0.79 (dcm) cut off > 1.27 (lvh) [83]	2D or anatomical M-Mode or by 3D volumetry	Yes—under certain conditions	To detect dilative or hypertrophic cardiomyopathy
Ees—LV elastance (mmHg/ml) (formula see [17])	7–20 cut off < 20 [72]	Blood pressure measurement and verifiable LV volume measurements are necessary	Optional—but helpful to detect subclinical states of cardiac diseases	To detect LV stiffness—especially in symptomatic patients with normal LV geometry or increased LV wall thickness
Ea—arterial elastance (mmHg/ml) (formula see [17])	− 4 to − 14 cut off > − 14 [72]	Blood pressure measurement and verifiable LV volume measurements are necessary	Optional—but helpful to detect subclinical states of cardiac diseases	To detect arterial stiffness (and see above)
MAPSE (mm)	♂ 10–18 cut off > 8 ♀ 8–20 cut off > 8 [82]	Assessment is possible by postprocessing of 2D cineloops of the 4ChV, not useful in MR and septal or lateral myocardial infarction	Optional—but helpful to detect subclinical states of cardiac diseases	To detect reduced longitudinal LV function, increased LV stiffness, and early stages of systolic dysfunction
MAPSE/TAPSE	♂ 0.45–0.81 cut off > 0.45 ♀ 0.36–1.00 cut off > 0.36 [82]	Assessment is possible by postprocessing of 2D cineloops of the 4ChV, not useful in MR, TR and septal or lateral myocardial or right ventricular infarction	Optional—but helpful to detect subclinical states of cardiac diseases	To detect LV fibrosis and interventricular abnormal mitral and tricuspid annular motion
** GLS (%)—global longitudinal strain**	**♂** − **17–27** **cut off > ** − **17** **♀–18–28** **cut off > -18** [87]	**LV strain analysis is only reliable in patients with adequate image quality. Avoid artefact tracking due to minor image quality**	**Yes**	**To detect reduced longitudinal LV function and pathological LV strain patterns at early stages**
GWI (mmHg%)—global work index	♂ 1270–2428 cut off > 1270 ♀ 1310–2538 cut off > 1310 [86]	Blood pressure measurement and respective image quality for LV strain analysis are prerequisites	Optional—but helpful to detect subclinical states of cardiac diseases	To distinguish between CAD and HHD
GCW (mmHg%)—global constructive work	♂1650–2807 cut off > 1650 ♀ 1543–2924 cut off > 1543 [86]	Blood pressure measurement and respective image quality for LV strain analysis are prerequisites	Optional—but helpful to detect subclinical states of cardiac diseases	To distinguish between CAD and HHD. To detect asynchrony
GWW (mmHg%)**—**global wasted work	♂ 94–271 cut off < 238 ♀ 74–278 cut off < 239 [86]	Blood pressure measurement and respective image quality for LV strain analysis are prerequisites	Optional—but helpful to detect subclinical states of cardiac diseases	To distinguish between CAD and HHD. To detect asynchrony
GWE (mmHg%)**—**global work efficiency	♂ 88–97 cut off > 88 ♀ 90–97 cut off > 90 [86]	Blood pressure measurement and respective image quality for LV strain analysis are prerequisites	Optional—but helpful to detect subclinical states of cardiac diseases	To distinguish between CAD and HHD. To detect asynchrony

For each echocardiographic parameter the normal ranges (if age dependent for elderlies more than 60 years) (and cut offs), methodological aspects, the importance of its determination, and the value to determine it in routine are listed. Mandatory parameters to be determined in clinical practice are labeled in bold print

*LVEDD* left ventricular end diastolic diameter, *LVESD* left ventricular end systolic diameter, *LVEDV* left ventricular end diastolic volume, *LVESV* left ventricular end systolic volume, *LVSV*_*tot*_ total left ventricular stroke volume, *LVSV*_*eff*_ effective left ventricular stroke volume, *LVOT* left ventricular outflow tract, *D*_*LVOT*_ diameter of the LVOT, *CO* cardiac output, *VTI*_*LVOT*_ velocity time integral determined in the LVOT, *LVEF* left ventricular ejection fraction, *BSA* body surface area, *EDLVs* enddiastolic septal LV wall thickness, *EDLVp* enddiastolic posterior LV wall thickness, *RWT* relative wall thickness, *LVRI* left ventricular remodeling index, *Ees* LV elastance, *Ea* arterial elastance, *MAPSE* mitral annular plane systolic excursion, *TAPSE* tricuspid annular plane systolic excursion, *GLS* global longitudinal strain, *GWI* global work index, *GCW* global constructive work, *GWW* global wasted work, *GWE* global work efficiency

**Fig. 8:**
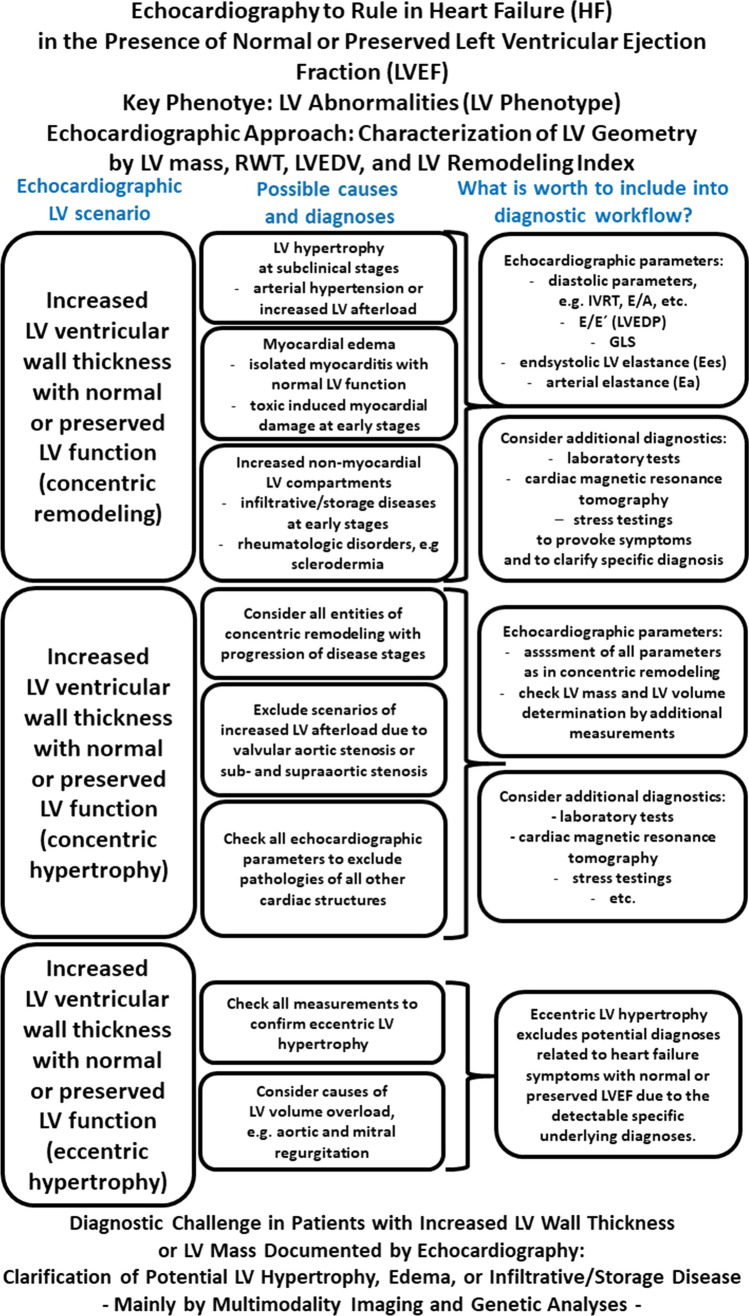
Scheme of the echocardiographic workflow in patients with “HFpEF” symptoms to characterize the echocardiographic LV phenotype and to exclude normal cardiac phenotype

﻿﻿Figure 8 summarizes the echocardiographic workflow in patients with “HFpEF” symptoms presenting normal cardiac phenotype or LV phenotype.

## The LA phenotype: abnormalities of left atrial (LA) volumes and LA function (diastolic dysfunction and increased LV filling pressures)

Increased stiffness of the LV wall impedes LV filling and increases LA pressure. This leads to the distinct morphological and functional LA alterations: the “LA phenotype” (Fig. 2).

**Fig. 9:**
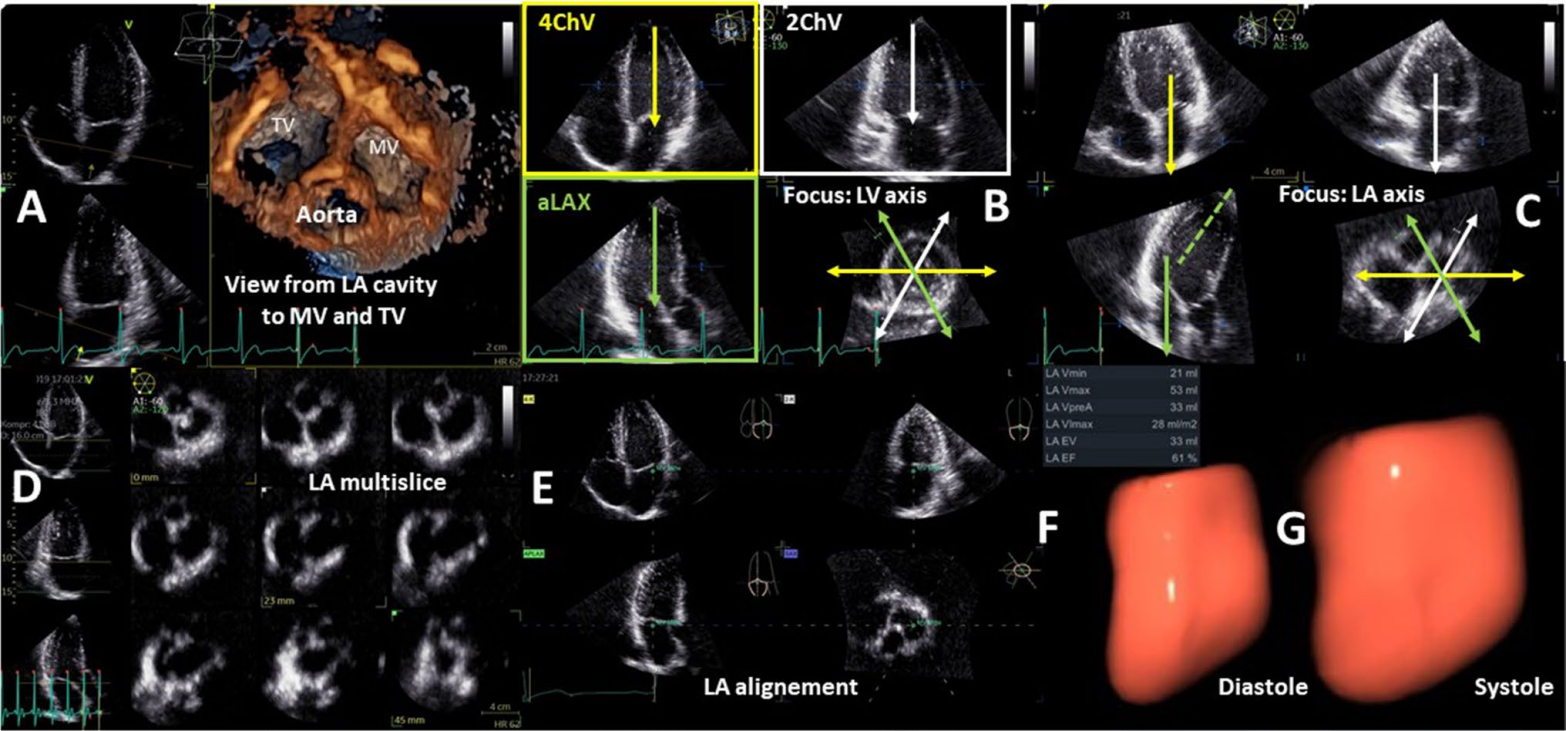
Illustration about the necessity for 3D-LA assessment. A presents the enface view from the LA cavity to the MV and TV. In B the apical 3D views (4ChV, 2ChV—apical 4- and 2-chamber view; aLAX—apical long axis view) within the 3D data set are centered to the LV illustrating the axis deviation of the LA mainly in the aLAX. In C the LA long axis is centered to document the deviation of LV an LA center line. In D a multisclice presentation of the 3D data set illustrate the complete acquisition of the LA volume. In E the LA alignment for LA volume determination is shown. In F the diastolic LA volume, in G the systolic LA volume is presented by a 3D volume calculation

The chronic increase in LA pressure results in progressive LA dilatation with increased volumes. Traditionally, LA size is measured in the anterior–posterior dimension from parasternal views. However, in pathologic states the LA frequently enlarges predominantly in the long-axis direction (base-apex). Therefore, the assessment of LA volume during pathological conditions should at least be performed by biplane planimetry from apical views. Volumetry of the complete LA by 3D TTE is preferred because centering of the longitudinal LA dimensions in standardized apical LV views may lead to foreshortening and systematic over- or underestimation of LA volumes when compared to 3D data sets (Fig. 9).

Biplane LA volume measurement indexed to body surface area (BSA) is the currently recommended standard. The obtained LA volume index (LAVI) has been associated with cardiovascular outcomes [33]. A maximum LAVI > 34 ml/m^2^ documents an increased LA volume [2, 4, 7, 33, 35, 48]. Different echocardiographic parameters of LA function can be described by conventional LA volume measurements: the maximum LA volume (LAV_max_) measured at LV end systole (onset of MV opening), the minimum LA volume (LAV_min_) at LV end diastole (prior to MV closure), and the LA volume prior to LA contraction at the onset of P wave (LAV_preA_). The echocardiographic parameters characterizing LA function and the respective calculations are listed in Table 2 [53, 89].

**Table 2:** Echocardiographic parameters characterizing LA function and their calculations by conventional LA volume and LA strain determination

Echocardiographic parameter	LA function (LA strain)	Calculation (by volumes)
Total LA emptying fraction (%) Normal range: 63–71 [53]	Global LA reservoir function Normal range: 36.1 to 48.0 [89, 91]	(LAV_max_ − LAV_min_)/LAV_max_
Expansion index Normal range: 171–250 [53]	Reservoir Index (no strain data available) [91]	(LAV_max_ − LAV_min_)/LAV_min_
Passive LA emptying fraction (%) Normal range: 38–49 [53]	LA conduit function Normal range: 20.4 to 31.8 [89, 91]	(LAV_max_ − LAV_preA_)/LAV_max_
Active LA emptying fraction (%) Normal range: 35–48 [53]	LA booster pump function Normal range: 12.9 to 19.5) [89, 91]	(LAV_preA_—LAV_min_) / LAV_preA_

*LA*—left atrial, *LAV*_max_ maximum LA volume, *LAV*_min_ minimum LA volume

Of all LA volume measurements, maximum LAVI (LAVI_max_) has the most important prognostic value [90–92]. However, minimum LAVI (LAVI_min_) might be better to characterize LA function during sinus rhythm because it may better reflect elevated LVEDP and PCWP than LAVI_max_ [93, 94]. The main practical challenge to assess LAVI_min_ is the higher error-proneness in comparison to LAVI_max_ due to inappropriate LA centering in the respective sectional 2D planes [91, 95–97]. The geometric assumptions of the LA size by 2D echocardiography usually cause an underestimation of LA volumes in comparison to LA volume measurements by 3D echocardiography [53, 91, 97, 98] underlining the necessity of 3D echocardiography for a better LA volume assessment. With respect to 3D echocardiography a higher threshold for the maximum LAVI above 40 ml/m^2^ has been proposed (instead of 34 ml/m^2^ by 2D) [53, 91, 97, 98]. In chronic AF threshold for maximum LAVI are higher than a LAVI_max_ of 40 ml/m^2^ in the literature [2, 91].

LA function can presumably be more objectively assessed by analysis of longitudinal LA strain. However, atrial strain is to a large extent determined by ventricular strains and modulated by the atrial and ventricular volumes. LA strain (LAS) is analyzed using apical 2D four-chamber views by triggering the beginning of the QRS complex [53]. Global average LA reservoir (LASr), LA conduit (LASr minus average LAS value at the onset of the p-wave), and LA contraction strain (LASct = LASr − LA conduit strain) are extracted from the respective strain curves. In patients with atrial fibrillation, LAS analysis was limited to the investigation of LASr and LAScd, as proposed by the recent EACVI recommendations [53, 85, 89, 90].

**Fig. 10:**
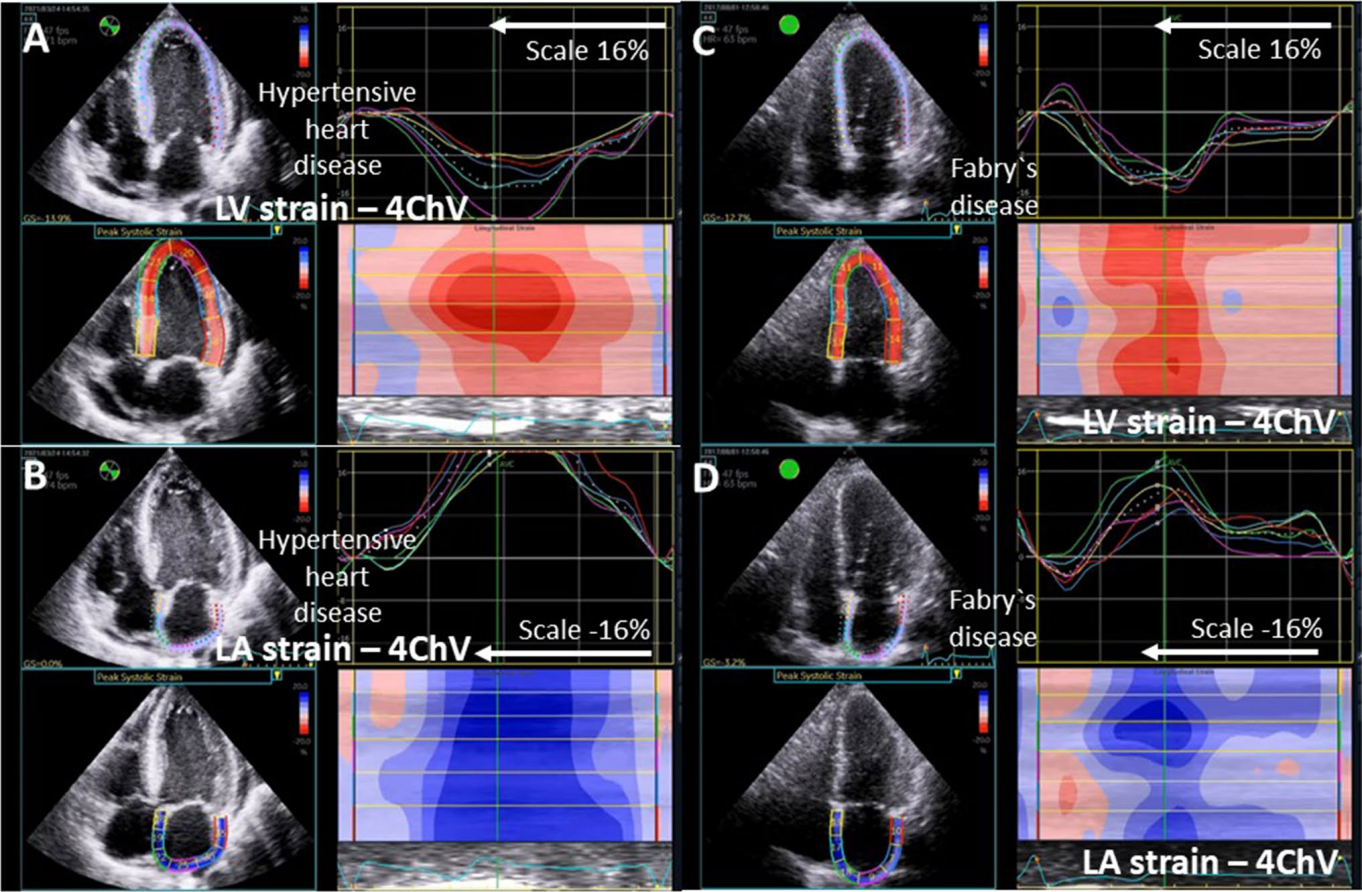
Illustration about advanced strain assessment in patients with “HFpEF” symptoms. In A, a quad screen of 4ChV (4-chamber view) documents LV strain analysis by presenting the tracking LV area, the respective strain graphs of the regional LV strain, the corresponding maximum strain values, and the color-coded curved strain M-Mode in a patient with hypertensive heart disease. Reduced mid-basal strain and normal mid-apical strain values are documented. In B, a comparable quad screen of the 4ChV for LA strain analysis is shown documenting LA strain values higher than 16%. In C, a comparable quad screen of 4ChV for LV strain analysis in a patient with Fabry’s disease illustrates reduced reginal LV strain values in all LV segments. In D, a comparable quad screen of 4ChV for LA strain analysis in the Fabry patient disease illustrates the possibility of early detection of LA dysfunction by the reduced LA strain values

LAS provides valuable additive information for the differential diagnosis between infiltrative/storage diseases like amyloidosis, hypertrophic cardiomyopathy and other types of LA and LV wall thickening including edema [99–101] (Fig. 10). Two distinct mechanisms can theoretically lead to LA dysfunction in infiltrative/storage diseases: firstly, the increased LA pressure induced by LV dysfunction and secondly, the direct accumulation of pathological deposits in the LA wall. Thus, hypertrophic cardiomyopathy as a primary LV disease affects LA dysfunction to a lesser degree than e.g. infiltrative/storage diseases. The diagnostic strength of LAS has been shown to be better than classical LV strain features like “apical sparing” [99–101]. Recent data suggests that LA dysfunction plays an essential role in the clinical course of patients with “HFpEF” symptoms due to early stages of infiltrative/storage diseases followed by DD and AF [99–101].

The clinical symptoms and the impaired exercise capacity in patients with “HFpEF” symptoms can mainly be explained by DD due to increased LVEDP at rest and/or during exercise. Historically, DD has initially been graded mainly according to the pw transmitral inflow E/A ratio. A high E/A ratio ≥ 2 with a short E-wave deceleration time (Edt) < 140 ms indicates severely impaired diastolic function due to increased LVEDP, however rarely documented at compensated stages. The frequently observed E/A ratio < 0.8–1 with a normal or prolonged Edt can be classified as abnormal relaxation and may indicate a slightly elevated LVEDP with normal LA pressure and no evidence for pulmonary congestion at rest [102–104]. However, the diagnostic value of the E/A ratio is severely limited by its bimodal behavior with the inability to distinguish between a normal and elevated filling pressure if the E/A is between 0.8 and 2. This limitation may be overcome by integrating pw Doppler information from pulmonary venous inflow. The duration of atrial reversal flow in the pulmonary vein is longer than the duration of forward transmitral atrial flow (A-wave) in the presence of elevated LVEDP [102–104]. Elevated LV filling pressures will also progressively shorten isovolumic relaxation time (IVRT) < 60–80 ms, which is typically obtained by a cw Doppler placed in-between the transmitral inflow and LV outflow [105]. The recommended and more practical alternative to estimate LVEDP can be performed by tissue Doppler imaging of the E´ wave in the basal septal and lateral LV segments. An average E/E’ ratio > 15 calculated with the mean E´ velocity of the basal septal and lateral LV segments indicates a significantly elevated LVEDP and is a major criterion in the HFA-PEFF score. However, several coexisting conditions such as the presence of left bundle branch block, valvular regurgitation, regional wall motion abnormalities affect the E/E’ ratio. If E/E´ratio is measured within the grey zone between 9 to 14, further echocardiographic parameters can be determined to estimate the degree of DD [4, 34, 41, 55, 84, 102–107]. The elevated LVEDP is transmitted through the pulmonary vasculature and will chronically lead to an increase in pulmonary pressure with mild to moderate postcapillary pulmonary hypertension. This can be identified by an increase in the tricuspid peak regurgitant (TR) velocity > 2.8 m/s reflecting an estimated systolic pulmonary artery pressure (sPAP) > 35 mmHg. These hemodynamic results may importantly vary with changing volume conditions.

All echocardiographic parameters defining DD are changing with age. An age-related decrease in the E/A ratio, mitral annular velocities, septal and lateral E’ has been observed in many population-based studies [108–110]. Also, mitral annular velocities decrease significantly during a lifetime. Elevated LV filling pressures and DD are not synonymous but refer to abnormal mechanical LV properties. Thus, patients with “HFpEF” symptoms usually have a higher sPAP, E/E’ ratio, and LAVI than patients with LV wall thickening presumably due to arterial hypertension without HF signs [111].

Patients with “HFpEF” symptoms and sole LA abnormalities are rare.

Table 3 summarizes the echocardiographic parameters, which are important to characterize the LA phenotype. The parameters are listed including normal ranges and cut offs [84, 85, 89, 102, 103, 110–113], methodological aspects of TTE determination, their importance to be determined, and their reasons, why it is worth to determine the respective parameter in clinical routine. Specific parameters can be helpful to detect functional LA abnormalities—especially in subclinical stages of possible cardiac diseases.

**Table 3:** Echocardiographic parameters characterizing patients with heart failure (HF) symptoms and normal or preserved left ventricular ejection fraction (LVEF) in left atrial (LA) phenotype

Echocardiographic parameter	Normal ranges—cut offs	Methodological aspects	Mandatory to determine (methods)	Why worth to do in routine
LA parameters and parameters of diastolic function				
LAD—left atruial diameter (mm)	♂ 31–39 Cut off < 39 ♀ 28–37 Cut off < 37 [85]	“Old” parameter, which can only be used in normal LA geometry	No—only if LA dimensions are documented as normal	To document LA dimension—if LA geometry is normal
LAD/BSA (mm/m^2^)	♂ 13–23 Cut off < 23 ♀ 14–24 Cut off < 24 [85]	“Old” parameter, which can only be used in normal LA geometry	No—only if LA dimensions are documented as normal	To document LA dimension—if LA geometry is normal
**LAVI**_**max**_**—maximum LA volume indexed to BSA (ml/m**^**2**^**)**	**♂ 18–35** **Cut off < 39** **♀ 18–36** **Cut off < 38** **(Cut off < 34)** [84, 85, 112]	**Avoid foreshortening, prefer triplane analysis or 3D volumetry. Increased LA volume predicts increased LV filling pressure**	**Yes**	**To document chronic diseases due to impaired LV filling**
LAVI_min_—minimum LA volume indexed to BSA (ml/m^2^)	♂ 8–18 Cut off < 18 ♀ 18–18 Cut off < 18 [6]	Avoid foreshortening, prefer triplane analysis or 3D volumetry. Increased LAVI_min_ predicts impaired active LA contractility	Yes—under certain conditions	To document impact on active LA contraction on global LA function
Total LA emptying fraction (LAEF) (%)	51–61 Cut off > 38 [112]	Avoid foreshortening, prefer triplane analysis or 3D volumetry. Reduced LA emptying fraction indicates LA dysfunction	Yes-—under certain conditions	To characterize LA function
** Average LA reservoir strain—reservoir LAS (%):** **LA reservoir function**	**31–42** **Cut off > 23** [89]	**LA strain analysis is only possible if image quality is adequate. 4ChV is usually used for LA strain analysis**	**Yes—especially in normal or LV phenotype—helpful to detect subclinical states of cardiac diseases**	**To characterize global LA function**
Passive LA conduit strain—passive LAS (%): LA conduit function	15–23 Cut off > 11 [89]	LA strain analysis is only possible if image quality is adequate. 4ChV is usually used for LA strain analysis	Yes—especially in normal or LV phenotype—helpful to detect subclinical states of cardiac diseases	To characterize passive LA filling properties
Active LAS contraction strain—active LAS (%): LA contraction function	14–21 Cut off > 8 [89]	LA strain analysis is only possible if image quality is adequate. 4ChV is usually used for LA strain analysis	Yes—especially in normal or LV phenotype—helpful to detect subclinical states of cardiac diseases	To characterize active LA contractility
LA stiffness—E/E´ devided by LAEF (%^−1^)	0.13–0.17 Cut off < 0.27 [89]	Standardize the Doppler assessment with respect to breathing to ensure comparability in follow-ups	No—especially in normal or LV phenotype—helpful in suspected infiltrative/storage diseases	To detect causes of LA stiffness by conventional parameters
LA stiffness—E/E´ devided by LAS (%^−1^)	0.18–0.29 Cut off < 0.55 [89]	Standardize the Doppler assessment with respect to breathing to ensure comparability in follow-ups	No—especially in normal or LV phenotype—helpful in suspected infiltrative/storage diseases	To detect causes of LA stiffness using speckle tracking
** E-peak E-wave velocity (cm/sec)**	**♂ 42–116** **Cut off > 42** **♀ 43–115** **Cut off > 43** [110]	**Acquire the pw Doppler spectra using Duplex mode to control correct positioning of the sample volume at the level of mitral valve (MV) coaptation**	**Yes—to differentiate between normal, abnormal relaxation, pseudo-normal, and restrictive**	**E reflects LA-LV gradient during early diastole**
** Peak A-wave velocity (cm/sec)**	**♂ 25–93** **Cut off > 25** **♀ 29–93** **Cut off > 29** [110]	**Acquire the pw Doppler spectra using Duplex mode to control correct positioning of the sample volume**	**Yes—to differentiate between normal, abnormal relaxation, pseudo-normal, and restrictive**	**A reflects LA-LV gradient during late diastole**
** Transmitral A-duration (msec)**	**100–176** **Cut off > 100** [102, 103]	**Acquire an additional pw Doppler spectrum of blood LV inflow at the level of mitral anulus (MA). Time speed must be100mm/sec to ensure sufficient temporal resolution**	**Yes—especially in normal or LV phenotype**	**To be able to compare forward and retrograde LA blood flow during LA contraction**
** Transmitral E/A ratio**	**♂ 0.62–2.34** **cut off > 0.62** **♀ 0.32–2.44** **cut off > 0.32** [110]	**Acquire the pw Doppler spectrum with sharp contours (possibly highest Doppler frequencies) with a sample volume in the central blood stream of LV inflow**	**Yes—- to differentiate between normal, abnormal relaxation, pseudo-normal, and restrictive**	**To distinguish between impaired LV relaxation, pseudonormal conditions, and LV restriction**
** Edt—E-wave deceleration time (msec)**	**♂ 78–302** **cut off > 78** **♀ 99–275** **cut off > 99** [110]	**Acquire the pw Doppler spectrum with sharp contours (possibly highest Doppler frequencies) with a sample volume in the central blood stream of LV inflow**	**Yes- especially in normal or LV phenotype**	**To detect impaired LV relaxation and LV stiffness**
** IVRT—isovolumetric relaxation time (msec)**	**73–101** **cut off ≤ 70** [102, 103]	**Acquire an additional pw Doppler spectrum with the sample volume positioned at the anterior mitral leaflet. IVRT estimates relaxation (τ)**	**Yes—especially in normal or LV phenotype**	**Prolonged in impaires relaxation; shortened if LAP increases**
L-wave (transmitral velocity spectrum and tissue doppler spectra)	Qualitative sign of diastolic dysfunction [102, 103]	The L-wave is documented in the transmitral pw Doppler spectrum and in the LV tissue Doppler spectra	Yes	If present, indicates increased LVEDP
** Peak E´-velocity basal septal (cm(sec)**	**♂ 6–11** **Cut off > 6** **♀ 5–10** **Cut off > 5** [110]	**Acquire the pw tissue Doppler spectra using Duplex mode to control proper sample volume positioning of. Try to center the LV septum for optimal image quality**	**Yes**	**E´ includes LV relaxation, restoring forces and LV filling pressure**
** Peak E´-velocity basal lateral [cm (sec)]**	**♂ 5–16** **Cut off > 5** **♀ 5–14** **Cut off > 5** [110]	**Acquire the pw tissue Doppler spectra using Duplex mode to control proper sample volume positioning. Try to center the later LV segment for optimal image quality**	**Yes**	**E´ includes LV relaxation, restoring forces and LV filling pressure**
** Average E/E´ratio**	** < 8 normal** **8–14 borderline** ** > 14 pathological** [102, 103]	**Check the respective positions of the sample volumes and standardize both documentation to comparable breathing periods**	**Yes**	**To estimate LVEDP. E/E’reflects normal or pathological relaxation**
T_E´-E_—time interval between E´- and E-onset (msec)	0–8 Cut off > 8 [113]	The estimation is within the limit of detection. Significant differences of time intervals in Doppler spectra can be detected by intervals > 20 ms	Optional—it can be used as a qualitative sign of diastolic dysfunction	T_E´-E_ can distinguish between restriction (prolonged) and constriction (normal)
IVRT/T_E´-E_ ratio	> 2 [113]	The estimation should only be performed if T_E´-E_ is > 20	Optional—but helpful to detect increased LVEDP	If ratio is < 2, PCWP and LAP are increased
ArD—retrograde pulmonary vein Ar-duration (msec)	53–173 Cut off Ar < A [102, 103]	Acquire pw Doppler spectrum using low pulse repetition frequency (LPRF). Prefer time speed of the spectrum at 100 mm/sec or more to ensure sufficient temporal resolution	Yes—especially in normal or LV phenotype	Prolonged ArD indicates diastolic dysfunction an increased VEDP
Peak Ar velocity (cm/s)	− 11to − 39 Cut off < 35 [102, 103]	Acquire pw Doppler spectrum using LPRF. Try to increase contour sharpness by increasing Doppler frequencies in the range of LPRF	Yes—especially in normal or LV phenotype	Increased Ar velocity indicates increased VEDP
Transmitral A duration–Ar duration	0–20 cut Off < 30 [102, 103]	For optimal documentation of sample volume position at the levels of MV coaptation and MA acquire both spectra using Duplex mode	Yes—especially in normal or LV phenotype	Prolonged A—Ar indicates increased LVEDP
Vp—left ventricular diastolic flow propagation (cm/sec)	Cut off ≥ 50 [102, 103]	Vp correlates with LV relaxation and the invasive parameter (τ. Adjust color Doppler setting and prefer time speed of the spectrum at 100 mm/sec or more	Yes—especially in normal or LV phenotype	Decreased Vp indicates LVEDP increase
E/Vp ratio (sec)	Cut off ≤ 2.5 [102, 103]	Perform Measure E and Vp in transmitral pw spectra and mitral flow color M-modes at standardized comparable breathing intervals	Yes—especially in normal or LV phenotype	E/Vp correlates with LAP and PCWP. Decreased E/Vp indicates increases of LAP and PCWP

For each echocardiographic parameter the normal ranges (and cut offs), methodological aspects, the importance of its determination, and the value to determine it in routine are listed. Mandatory parameters to be determined in clinical practice are labeled in bold print

*LAD* left atrial diameter, *BSA* body surface area, *LAVI*_max_ maximum LA volume indexed to BSA, *LA* left atrial, *LAVI*_min_ minimum LA volume indexed to BSA, *LAEF* total LA emptying fraction, *LAS* left atrial strain, *E* maximum early mitral flow velocity, *E´* maximum early tissue Doppler lengthening velocity of the myocardium near to the mitral anulus, *A* maximum forward transmitral atrial flow velocity, *Edt* E-wave deceleration time, *IVRT* isovolumetric relaxation time, *L* flow velocity peak of transmitral flow during diastasis, *T*_*E´-E*_ Time interval between *E´* and E-onset, *ArD* retrograde pulmonary vein flow duration, *Vp* left ventricular diastolic flow propagation

**Fig. 11:**
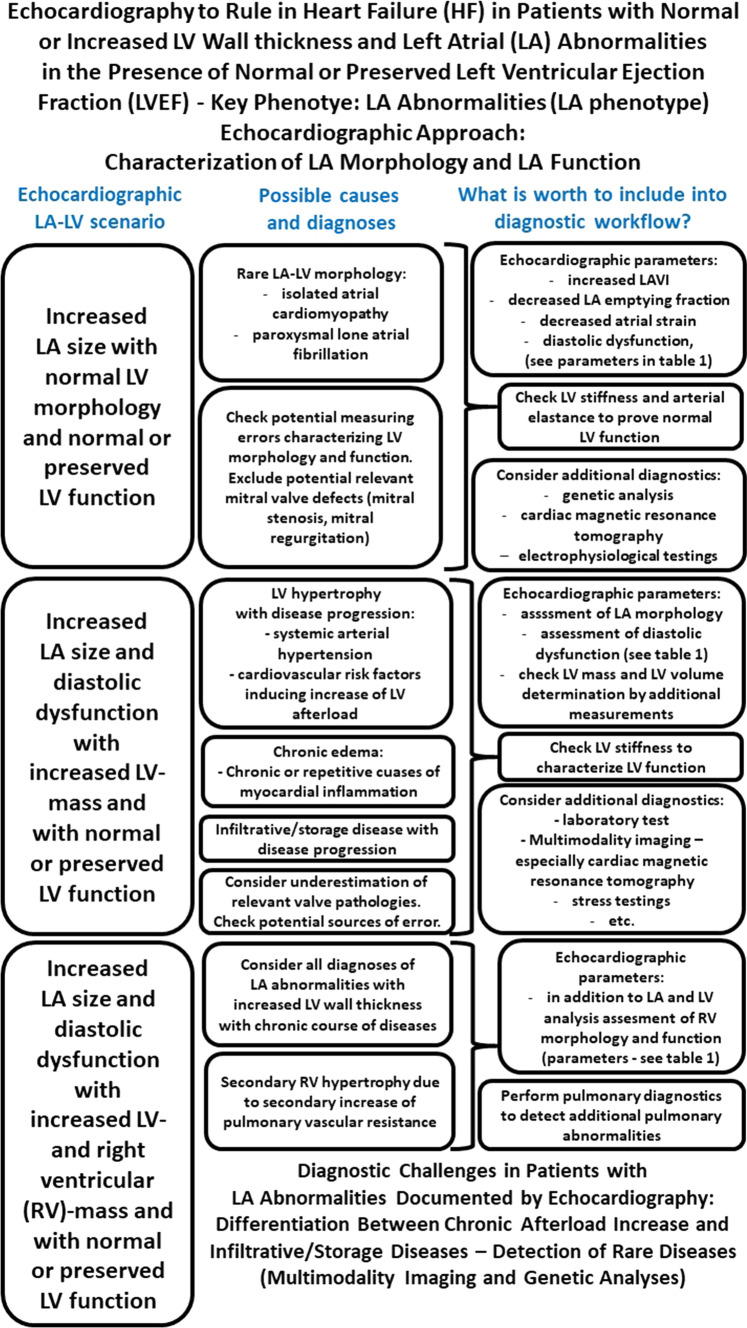
Scheme of the echocardiographic workflow in patients with “HFpEF” symptoms to characterize the echocardiographic LA phenotype

Figure 11 summarizes the echocardiographic workflow in patients with “HFpEF” symptoms presenting the LA phenotype.

## The RA/RV phenotype: abnormalities of right atrial (RA) volumes and RA function as well as of right ventricular (RV) volumes, RV function, and RV geometry

In patients with “HFpEF” symptoms abnormalities of RA and RV morphology and function can be induced by several factors. The constellation of pathological echocardiographic findings and its interpretation encompasses RV wall thickening, RA and RV volume, RV contractility and deformation as well as tricuspid and pulmonary valvular function to differentiate between RV volume and RV pressure overload [114, 115]. Increased RV contractility combined with increased RV volume primarily refers to compensated staged with increased RV volume load, increased RV contractility combined with RV wall thickening and normal RV volumes primarily refers to compensated stages with RV pressure load. Thus, these echocardiographic features can lead to diagnoses like tricuspid regurgitation or left–right shunts due to increased RV volume load or to diagnoses like primary or secondary pulmonary hypertension due to increased RV pressure load [114, 115]. The interpretation of the echocardiographic findings is more challenging and difficult at decompensated stages.

Due to secondary pulmonary hypertension or infiltrative/storage diseases the RA/RV phenotype is often linked to the LA phenotype and the LV phenotype. The isolated RA/RV phenotype in patients with “HFpEF” symptoms normally characterizes primary pulmonary hypertension due to specific lung diseases or rare diseases like arrhythmogenic RV dysplasia. Especially in this scenario, the non-invasive determination of pulmonary vascular resistance by echocardiography is recommended (Table 4). The combination of the RA/RV phenotype with the other echocardiographic phenotypes is observed in almost all other cardiac diseases.

**Table 4:** Echocardiographic parameters characterizing patients with heart failure (HF) symptoms and normal or preserved left ventricular ejection fraction (LVEF) in the combined right atrial (RA) and right ventricular (RV) phenotype

Echocardiographic parameter	Normal ranges—cut offs	Methodological aspects	Mandatory to determine (methods)	Why worth to do in routine
RV parameters				
** Enddiastolic RV free wall thickness (mm)**	**3–5** **Cutoff < 5** [124]	**Parasternal or subcostal short axis views of the RVOT can be used. Parasternal assessment is influenced by near field, subcostal assessment by far field**	**Yes**	**To detect or exclude RV hypertrophy** **Increased RV wall thickness indicates RV pressure overload**
FAC (%)—fractional area change of the RV (assessed by RV focused 4ChV)	Cut off < 35 [115]	Estimation is error prone to individual RV topography in relation to LV in the 4ChV. FAC can only be interpreted if no or only mild TR is present	No—usually too error-prone, only if 3D acquisition is not possible	To estimate RV size and RV function
** 3D RVEDV—RV enddiastolic volume (ml)**	**♂ 98–141** **Cut off > 98** **♀ 75–110** **Cut off > 75** [125]	**RV volumes should be measured by 3D echocardiography** **2D parameters (areas, diameter) are too error prone due to improper standardization of the RV in respective sectional planes**	**Yes—if image quality is adequate**	**To estimate RVEDV—especially in abnormal RV geometry**
**3D RVESV—RV endsystolic volume (ml)**	**♂ 37–67** **Cut off > 37** **♀ 27–49** **Cut off > 27** [125]	**RV volumes should be measured by 3D echocardiography** **2D parameters (areas, diameter) are too error prone due to improper standardization of the RV in respective sectional planes**	**Yes—if image quality is adequate**	**To estimate RVEF and total RVSV**
**3D RVSV**_**tot**_**—total RV stroke volume (ml)**	**♂ 53–83** **Cut off > 53** **♀ 42–67** **cut off > 42** [125]	**RV volumes should be measured by 3D echocardiography** **2D parameters (areas, diameter) are too error prone due to improper standardization of the RV in respective sectional planes**	**Yes—if image quality is adequate**	**To estimate RVEF and total RVSV, which corresponds to effective RVSV, if PV and TV are normal**
D_RVOT_ (mm)—diameter of the right ventricular outflow tract (RVOT)	♂ 17—27 ♀ 17—27 [33]	3D imaging is preferred with respect to verifiable standardization. Diameter of the pulmonary trunk can be used as an alternative	Yes—under certain coditions	To estimate RVSV_eff_ and cardiac output (CO)
Cardiac output (CO) determined by RVSV_eff_ (effective RV stroke volume) using pulsed waved (pw) Doppler x heart rate (ml/l) - LVSV_eff_ = 2 × D_RVOT_ x VTI_RVOT_ where VTI_RVOT_ is velocity time integral of flow velocities determined in the right ventricular outflow tract	♂ 3.5–8.2 ♀ 3.3–7.3 [88]	Proper positioning of the pw sample volume in relation to the D_RVOT_ is mandatory (if pulmonary trunc as an alternative is used, position of the sample volume must be adjusted—angulation of the cursor parallel to the blood stream is mandatory (subcostal imaging should be considered	Yes- under certain coditions	To estimate RVSV_eff_ and CO To compare LVSV_eff_ and RVSV_eff_
** 3D RVEF—RV ejection fraction (%)**	**♂ 54–59** **Cut off > 54** **♀ 56–62** **cut off > 56** [125]	**RV volumes should be measured by 3D echocardiography** **2D parameters (areas, diameter) are too error prone due to improper standardization of the RV in respective sectional planes**	**Yes—if image quality is adequate**	**To estimate normal or preserved LVEF**
RVEDV/BSA (ml/m^2^)	♂ 55 – 68 Cut off > 55 ♀ 48—60 Cut off > 48 [125]	RV volumes should be measured by 3D echocardiography 2D parameters (areas, diameter) are too error prone due to improper standardization of the RV in respective sectional planes	Optional—by 3D volumetry	To adjust LV volumes in extreme conditions
RVESV/BSA (ml/m^2^)	♂ 22–32 Cut off > 22 ♀ 19–15 Cut off > 19 [125]	RV volumes should be measured by 3D echocardiography 2D parameters (areas, diameter) are too error prone due to improper standardization of the RV in respective sectional planes	Optional—by 3D volumetry	To adjust LV volumes in extreme conditions
TAPSE (mm)	♂ 17–29 cut off > 17 ♀ 17–27 Cut off > 17 [82, 84]	Assessment is possible by postprocessing of 2D cineloops of the 4ChV, not useful in TR and shunts	Optional—but helpful to detect subclinical states of cardiac diseases	To detect RV dysfunction in the presence of normal TV or only mild TR
RV free wall peak pw S ‘ velocity (cm/sec)	♂ 8—19 cut off > 8 ♀ 9—17 Cut off > 9 [110]	Acquire the pw tissue Doppler spectra using Duplex mode to control proper sample volume positioning of. Try to center the RV free wall for optimal image quality	Optional—but helpful in the normal and atrial phenotype	To estimate RV contractility
RV-IVRT = Free wall RV total isovolumetric relaxation time by pw tissue Doppler	0–36 Cut off ≤ 36 [123]	Acquire the pw tissue Doppler spectra with time speed of 100 mm/sec	Optional—but helpful in the normal and atrial phenotype	To estimate RV relaxation
TVI RV free wall strain (%)	27–31 Cut off > 17 [115]	Acquire three consecutive RR-intervals to detect artefacts and drifting	Optional—but helpful in the normal and atrial phenotype	To characterize longitudinal RV deformation
RV free wall GLS (%)	25–32 Cut off > 18 [16]	Ensure the full myocardial tracking of all RV myocardial layers	Optional—but helpful in the normal and atrial phenotype	To characterize longitudinal RV deformation
RIMP by pw RIMP = (TCO–ET)/ET -right index of myocardial performance	0.21–0.43 cut off < 0.43 [114, 115]	Acquire transpulmonic and transtricuspid pw Doppler spectra at the same heart rate to be comparable	Yes—especially in the RV phenotype	Increased RIMP indicates RV dysfunction and indicates reduced filling and ejection intervals
RIMP by TVI RIMP = (IVRT + IVCT)/ET	0.22–0.54 cut off > 0.54 [114, 115]	Acquire the pw tissue Doppler spectra with time speed of 100 mm/sec. The isovolumetric time intervals can only be determined with high temporal resolution	Yes—especially in the RV phenotype	Increased RIMP indicates RV dysfunction and indicates reduced filling and ejection intervals
** Vmax**_**TR**_**–TR systolic peak velocity (m/sec)**	**Cut off ≤ 2.8** [114, 115]	**Vmax**_**TR**_** only reflects RV pressure if pulmonary stenosis is excluded and if RV is not decompensated**	**Yes**	**To estimate RVESP**
** sPAP = 4 × Vmax**_**TR**_^**2**^** plus estimated RAP (right atrial pressure)- estimated systolic pulmonary artery pressure (mmHg) -**	**Cut off ≤ 30** [114, 115]	**RV pressures can only be estimated if pulmonary stenosis is excluded and if RV is not decompensated**	**Yes**	**To estimate RVESP**
edVmax_PR_—PR end-diastolic peak velocity (m/sec)	No normal ranges in the literature—if normal RVEDP-values of 10–15 mmHg are assumed, cut off < 1.2 [114, 115]	Acquire a transpulmonic cw Doppler spectrum for assessment	Yes—especially in the RV phenotype	To estimate RVEDP (= dPAP)
dPAP = 4 × edVmax_PR_^2^ plus estimated RAP—estimated enddiastolic pulmonary artery pressure (mmHg)	No normal ranges in the literature, according to invasive assessment, normal dPAP < 10–15 mmHg [114, 115]	Acquire a transpulmonic cw Doppler spectrum for assessment	Yes—especially in the RV phenotype	To estimate RVEDP (= dPAP)
RAVI_max_ (ml/m^2^)	♂: Cut off < 30 ♀: cut off > 28 [7]	Estimation is error prone to individual RA topography in the 4ChV	No—usually too error-prone, only if 3D acquisition is not possible	To estimate RA dysfunction and chronic RV diseases due to increased RV filling pressures
** Collapse index inferior caval vein (%)**	**Cut off > 50** [115]	**Ensure centering of the inferior caval vein (VCI) during longitudinal scanning. Biplane scanning should be preferred to document the VCI simultaneously in a short axis view**	**Yes**	**To estimate right atrial pressure (RAP) and systemic congestion. Decreased values indicate RAP increase**
Pulmonary vascular resistance = PVR (msec^−1^) = (PEP/AcT)/(PEP + RVET), where PEP is preejection period (= time interval between TR onset and onset of systolic pulmonary flow), AcT is time interval between onset and peak pulmonary flow, and RVET is right ventricular ejection time	Cut off < 2.5 [114]	This index estimates PVR and correlates with wood units (WU). The methodological advantage is the robustness of Doppler time intervals	Optional—especially in RV phenotype	To estimate PVR
PVR (cm^−1^) = (10 × Vmax_TR_)/VTI_RVOT_, where VTI_RVOT_ is velocity time integral of flow velocities determined in the right ventricular outflow tract	Cut off < 2 [114]	This index estimate PVR and correlates with wood units (WU). Avoid methodological errors by acquiring the pw Doppler spectra. (adjust cursor and sample volume position)	Optional—especially in RV phenotype	To estimate PVR
PVR—(mmHg min cm^−1^) = sPAP/(heart rate x VTI_RVOT_)	Cut off > 0.076 [114]	This index estimates PVR. Increased index predicts severely increased PVR	Optional—especially in RV phenotype	To estimate PVR

For each echocardiographic parameter the normal ranges (and cut offs), methodological aspects, the importance of its determination, and the value to determine it in routine are listed. Mandatory parameters to be determined in clinical practice are labeled in bold print

*RV* right ventricular, *FAC* fractional area change of the RV, *4ChV* 4-chamber view, *RVEDV* RV enddiastolic volume, *RVESV* RV endsystolic volume, *RVSV*_tot_ total RV stroke volume, *RVOT* right ventricular outflow tract, *D*_*RVOT*_ diameter of the RVOT, *CO* cardiac output, *RVSV*_*eff*_ effective RV stroke volume, *VTI*_*RVOT*_ velocity time integral of flow velocities determined in the RVOT, *RVEF* RV ejection fraction, BSA body surface area, *TAPSE* tricuspid annular plane systolic excursion, S´- maximum systolic tissue Doppler velocity of the myocardium near to the tricuspid anulus at the RV free wall, *RV-IVRT* free wall RV total isovolumetric relaxation, *TVI* tissue velocity imaging, *RIMP* right index of myocardial performance, *TCO* time interval between tricuspid valve closure and opening, *ET* ejection time, *IVRT* isovolumetric relaxation time, *IVCT* isovolumetric contraction time, *TR* tricuspid regurgitation, *Vmax*_*TR*_ TR systolic peak velocity, *sPAP* systolic pulmonary artery pressure, *RAP* right atrial pressure, *PR* pulmonary regurgitation, *edVmax*_*PR*_ PR end-diastolic peak velocity, *dPAP* enddiastolic pulmonary artery pressure, *RAVI*_*max*_ maximum RA volume indexed to BSA, *VCI* inferior caval vein, *PVR* pulmonary vascular resistance, *PEP* pre-ejection period, *AcT* time interval between onset and peak pulmonary flow, *RVET* RV ejection time, *VTI*_*RVOT*_ velocity time integral of flow velocities determined in the RVOT

**Fig. 12:**
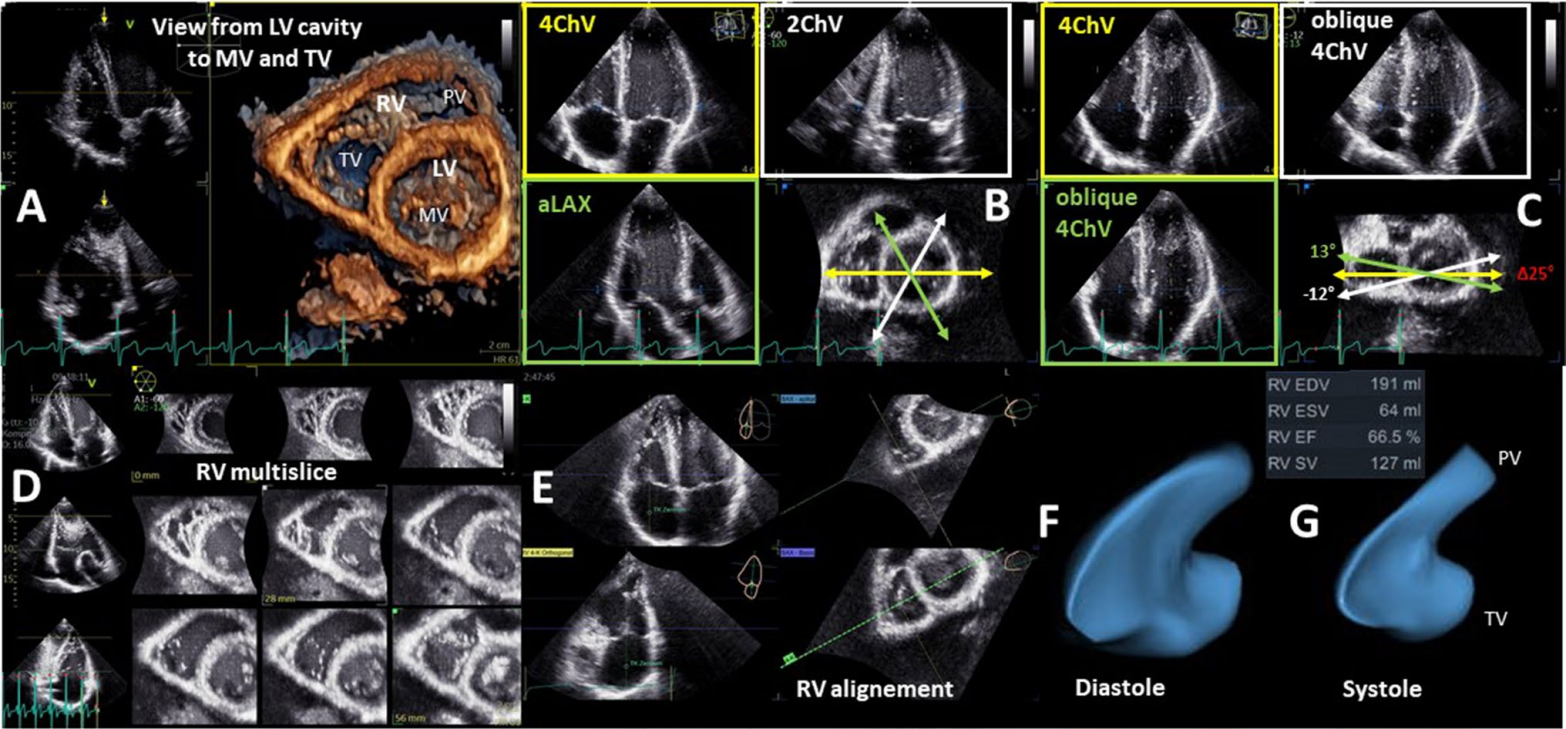
Illustration about the necessity for 3D-RV assessment—normal conditions. A presents the enface view from the LV cavity to the MV and TV. In B, the apical 3D views (4ChV, 2ChV—apical 4- and 2-chamber view; aLAX—apical long axis view) within the 3D data set are centered to the LV illustrating the standardized 4ChV by representative angular distance of 60° between aLAX, 2ChV, and 4ChV. In C, oblique 4ChV are shown to illustrate that even during normal conditions the deviation of non-standardized 4ChVs for 2D measurements is within a Δ of 25°. In D, a multisclice presentation of the 3D data set illustrate the complete acquisition of the RV volume. In E, the RV alignment for RV volume determination is shown. In F the diastolic RV volume, in G the systolic RV volume is presented by a 3D volume calculation

**Fig. 13:**
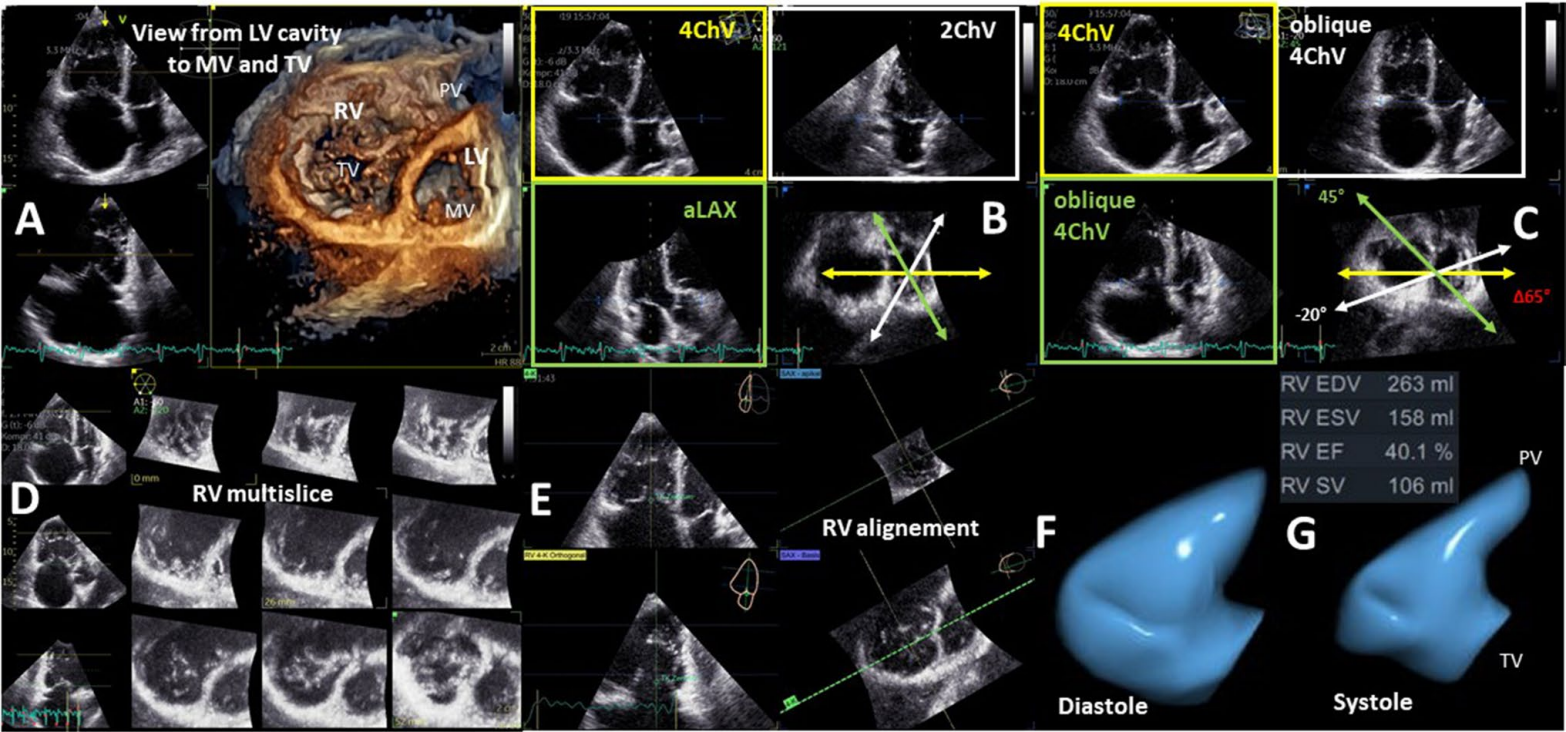
Illustration about the necessity for 3D-RV assessment -pathological conditions. A presents the enface view from the LV cavity to the MV and TV. In B the apical 3D views (4ChV, 2ChV—apical 4- and 2-chamber view; aLAX—apical long axis view) within the 3D data set are centered to the LV illustrating the standardized 4ChV by representative angular distance of 60° between aLAX, 2ChV, and 4ChV. In C oblique 4ChV are shown to illustrate that during pathological RV conditions the deviation of non-standardized 4ChVs for 2D measurements is within a Δ of 65°. In D a multisclice presentation of the 3D data set illustrate the complete acquisition of the RV volume. In E the RV alignment for RV volume determination is shown. In F the diastolic RV volume, in G the systolic RV volume is presented by a 3D volume calculation

The determination of the echocardiographic RV parameters presented in Table 4 should be considered if RV abnormalities are observed in patients with “HFpEF” symptoms [114, 115]. RV wall thickness, the tricuspid annular plane systolic excursion (TAPSE), sPAP, and RV strain are relatively robust parameters [33, 102, 103, 114, 115] whereas estimations of RV volumes resulting on 2D measurements are often error prone due to problems to standardize the sectional planes through the right ventricle [116–118]. Thus, 3D RV volume assessment is always recommended if possible—and 2D distance measurements to characterize RV volume should be avoided [33, 118]. Measurements of the RV fractional area change (FAC) determined by 2D planimetry of the end diastolic and end systolic RV area in an apical four chamber view focused to the right ventricle are often misleading due to methodology of standardization [116–118]. Thus, estimation of RV function should be performed by the assessment of RV ejection fraction (RVEF) by 3D echocardiography or by RV strain analysis (Figs. 12, 13).

RV strain should be measured in a RV-focused apical four chamber visualizing the RV free wall in the center of the scanning sector. Sector size should be minimized to increase frame rate for tissue Doppler measurements of the free RV wall [49, 50, 119]. Speckle tracking of the RV wall also like of the LV wall requires frame rates between 40 and 80/min. Then, RV strain can be analyzed in the basal, medial, and apical RV segments. In addition to RV strain, RA strain can be analyzed semi-automatically after manual determination of the endocardial borders. Global RA reservoir strain was assessed by analyzing the maximum excursion of the respective strain curve representing average peak strain values of all RA segments [50, 119, 120]. In addition, RA strain during passive RV filling (RA conduit strain) and during peak atrial contraction (RA active strain) can be determined. Comparable to LA strain RA conduit strain can be calculated by the difference of RA global strain and average RA strain value at the onset of the p-wave, whereas RA active strain can be calculated by the difference of RA global strain and RA reservoir strain. Reference values of mean RA reservoir strain, RA conduit and contraction strain are 44.9 ± 11.6%, 27.1 ± 9.5%, and 17.0 ± 5.9%, respectively [121, 122].

Table 4 summarizes the echocardiographic parameters, which are important to characterize the RA/RV phenotype. The parameters are listed including normal ranges and cut offs [33, 85, 87, 91, 113, 117, 118, 126–128], methodological aspects of TTE determination, their importance to be determined, and their reasons, why it is worth to determine the respective parameter in clinical routine. Specific parameters can be helpful to detect functional RA and RV abnormalities—especially in combination with pathological LA and LV findings.

**Fig. 14:**
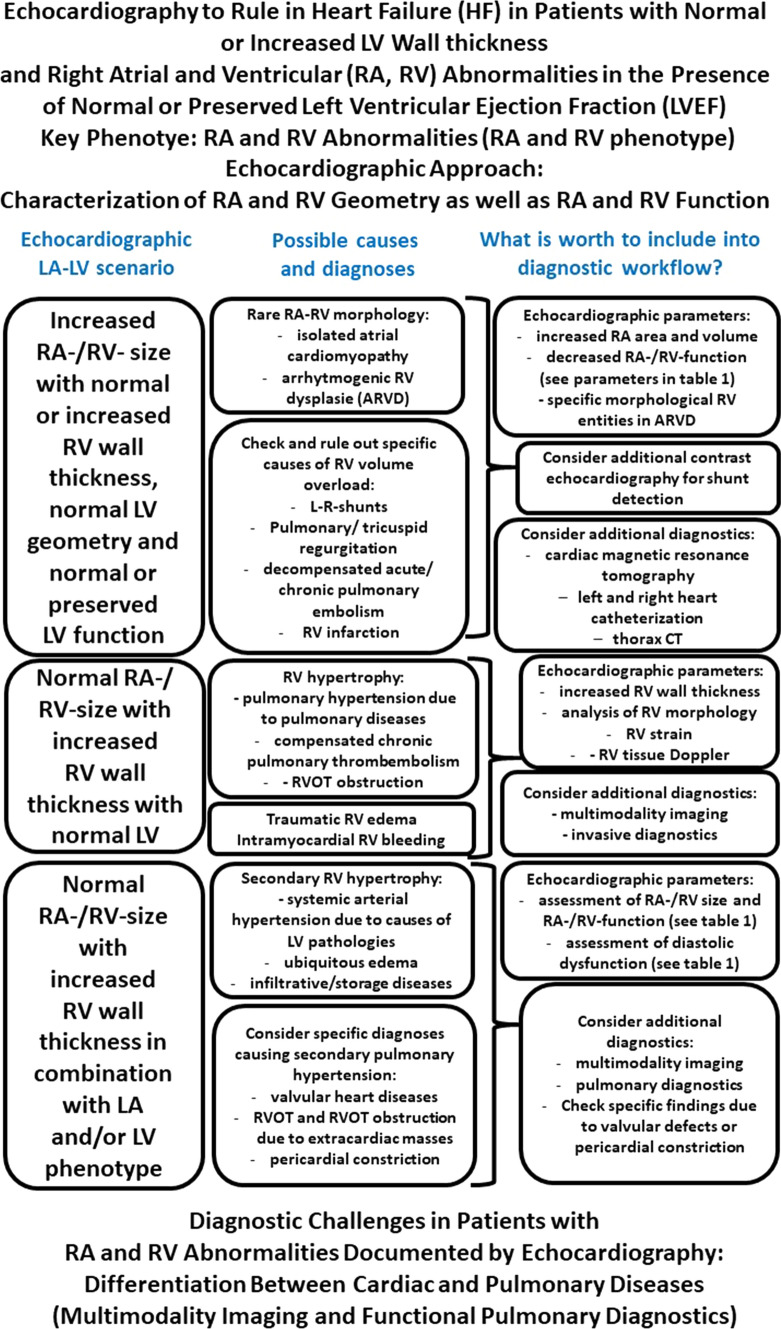
Scheme of the echocardiographic workflow in patients with “HFpEF” symptoms to characterize the echocardiographic RA/RV phenotype

Figure 14 summarizes the echocardiographic workflow in patients with “HFpEF” symptoms presenting the RA/RV phenotype.

## The role of diastolic stress echocardiography in patients with “HFpEF” symptoms

Sensitivity of resting echocardiographic parameters is limited to detect DD as the cause of “HFpEF” symptoms. Only 34%–60% of DD patients characterized by TTE have been confirmed with invasively proven DD in patients with preserved LVEF [62, 126]. Thus, artificial intelligence is proposed to predict HF in asymptomatic patients with normal cardiac phenotype, changes of diastolic filling properties and diastolic abnormalities with structural cardiac findings [127]. Thus, asymptomatic patients at rest might provide “HFpEF” symptoms due to DD during stress. The detection of DD development during physical diastolic stress echocardiography [2, 7] requires a proper standardization of the acquisition of Doppler spectra with respect to comparable breathing state and comparable sample volume positions at the respective stress levels. Besides these methodological challenges, increasing stress levels are normally limited due to the fusion of E- and A-wave with increasing heart rate. In consequence, DD detection by diastolic stress echocardiography should be confirmation by invasive hemodynamic exercise testing [7, 128, 129]. However, invasive hemodynamic studies are rarely performed to confirm an elevated PCWP in patients with “HFpEF” symptoms in clinical practice. Adding the criterion “exercise E/E’ > 14” to detect DD by diastolic stress echocardiography improved the sensitivity to 90%, but decreased specificity from 92 to 71% [126]. The hemodynamic response to exercise in patients with “HFpEF” symptoms was analyzed documenting that E/E’ at rest was higher in HF patients with small LV size and normal LA size group than in healthy controls, but that this difference did not reflect a difference in PCWP [128]. Furthermore, E/E’ did not significantly increase during stress in both groups and did not correlate with invasively measured PCWP at peak stress. It was concluded that increased arterial stiffness is presumably one of the key mechanisms of “HFpEF” symptoms [128, 129]. Stress testing in cardiology, however, is mainly used to detect coronary artery disease by stress-induced LV wall motion abnormalities, but it might obviously contribute to distinguish between cardiac and non-cardiac causes of “HFpEF” symptoms [2, 7].

## Summary

The present proposal provides an echocardiographic focused algorithm for patients with HF symptoms and preserved LVEF. In line with recent recommendations [2, 7], distinct cardiac phenotypes characterized by TTE are introduced as predominant gatekeepers for further diagnostic steps to clarify the specific underlying diagnoses. The algorithm starts with ruling out patients with non-cardiac causes for HF symptoms because edema has a low sensitivity of 20% and dyspnea on exertion a low specificity of 17% to be caused by cardiac failure [1]. In addition, a comparison of invasively measured PCWP with clinical HF classification according to the New York Heart Association (NYHA) showed in 61% (NYHA II) and in 14% (NYHA III) a normal PCWP < 16 mmHg, and biomarkers like NT-pro BNP showed no correlation to PCWP at rest [130]. Thus, symptoms suggestive of “HFpEF” may in some patients represent non-HF comorbidities” [131, 132]. The morphological classification of patients with “HFpEF” symptoms into the normal cardiac phenotype, the LV phenotype, the LA phenotype, and the combined RA/RV phenotype is the main challenge of the algorithm to detect the underlying pathologies responsible for “HFpEF” symptoms. The systematic echocardiographic approach emphasizes conventional, but uncommon parameters like Ees and Ea, but also modern parameters like RV volume, LA strain and myocardial work—determined by 3D echocardiography and deformation imaging—to improve echocardiographic detection of specific cardiac diagnosis in patients with “HFpEF” symptoms. The assessment of LA and LV stiffness might be particularly useful to detect subclinical pathologic entities in an apparently normal cardiac phenotype with borderline morphological abnormalities. For example, LA dysfunction might precede LA remodeling which is better predicted by LASr enables to distinguish asymptomatic patients with DD from those with HF symptoms [130]. Presumably, additional diastolic stress testing by dynamic stress echocardiography can further improve the diagnostic accuracy. In summary, the proposed diagnostic TTE workflow highlights the necessity to detect a specific diagnosis of non-cardiac and cardiac diseases. The common use of “HFpEF” as a *diagnosis* should be avoided.

## Conference discussion

*Prof. Dr. Andreas Hagendorff (Leipzig):* The “3. Mitteldeutscher Echokardiographie Kongress” in Leipzig had focused the topic “HFpEF” at the debate “focus on echocardiographic clinical trials: can we improve the HFA-PEFF algorithm by echocardiography? “and at the scientific session “heart failure with preserved or normal ejection fraction: a special echocardiographic challenge”. As in 2020 the congress in 2021 and the discussions about this expert proposal were again web based. To introduce into the following virtual conference discussion the president of our working group will start:

Please, tell us about the main diagnostic problem in patients with “HFpEF” symptoms in echocardiography.

*Dr. Andreas Helfen (Lünen):* According to recent recommendations the echocardiographic characterization of patients with “HFpEF” symptoms is mainly based on clinical symptoms, normal LV size and increased E/E´ as a surrogate parameter of increased LVEDP. However, it is well known that non-cardiac as well as cardiac diseases can be the underlying causes of the described symptoms. Thus, the multiple non-cardiac diseases with symptoms of reduced exercise capacity, edema and dyspnea on exertion should not be integrated into the definition of the “HFpEF” symptoms because cardiac morphology and function as well as PCWP can be within normal ranges in these patients. Thus, heart failure does not exist as the basic problem in this cohort. The uncertainty to clearly define patients with “HFpEF” symptoms according to the described criteria is potentiated by the increased incidence of the clinical symptoms with aging and obesity.

*Prof. Dr. Andreas Hagendorff (Leipzig):* How can we untangle the Gordian knot of “HFpEF” symptoms?

*Dr. Stephan Stöbe (Leipzig):* The answer runs like a common thread through the expert proposal. It has consequently been avoided to use “HFpEF” as a diagnosis. “HFpEF” is definitively not a diagnosis. It can only describe a syndrome or a symptom complex, which can be caused by several different pathologies. We need to focus on the specific underlying diagnoses in patients with “HFpEF” symptoms which is the central take home message of the expert proposal.

*Prof. Dr. Andreas Hagendorff (Leipzig):* Please explain the rationale to avoid the diagnosis “HFpEF” using an example.

*Prof. Dr. Dariusch Haghi (Ludwigshafen):* The increase of LV wall thickening is an excellent example to explain the importance of a specific diagnosis. LV wall thickening can be induced by hypertrophy of the myocardial fibers i. e. in hypertensive heart disease, aortic valvular stenosis, or hypertrophic cardiomyopathy, by edema i. e. in myocarditis and thoracic trauma, or by accumulation of pathological deposits i. e. in amyloidosis, Gaucher’s or Fabry’s disease. All these specific diagnoses require specific treatment and have different prognoses. Thus, we cannot put all these patients in one cohort to analyze therapeutic effects. It is like using cough as a diagnosis instead of a symptom. Cough due to lung cancer obviously needs another treatment than cough due to neuroses.

*Prof. Dr. Andreas Hagendorff (Leipzig):* How to improve diagnostic accuracy by echocardiography in patients with “HFpEF” symptoms? Which echocardiographic parameters are helpful?

*Priv.-Doz. Dr. Ole Breithardt (Kassel):* Echocardiography—especially TTE—offers a plenty of parameters which can be measured. However, a camel is a horse designed by committee. Thus, we must focus on conventional and presumably on a few modern parameters to characterize patients with “HFpEF” symptoms. In the tables we listed the echocardiographic parameters concerning the “HFpEF” scenario almost completely. However, we strongly recommend—of course—not all of them in clinical practice. But how to use uncommon parameters—when applicable under special conditions—if they are not described. Thus, the tables of the expert proposal are informative, helpful and provide orientation.

*Prof. Dr. Andreas Hagendorff (Leipzig):* Do you think the classification into cardiac phenotypes is useful?

*Dr. Jan Knierim (Berlin):* The characterization of cardiac morphology and function during real life conditions is the strength of echocardiography. Thus, it is reasonable to start with a descriptive approach of cardiac morphology and function when performing echocardiography. A lot of parameters are age- and gender-dependent. These and further factors must be integrated in a clinical and pathophysiological interpretation of the findings determined by a comprehensive echocardiography. Based on a structured echocardiographic investigation it might be possible to be more successful to detect specific diagnoses.

*Prof. Dr. Andreas Hagendorff (Leipzig):* This might be especially the problem in patients with “HFpEF” symptoms with the normal cardiac phenotype. How to handle this problem?

*Dr. Roland Brandt (Bad Nauheim):* This is definitively the most difficult issue in these patients, because either we have overlooked marginal pathological cardiac findings, or the patient has non-cardiac reasons for the symptoms. This scenario reflects the border between general screening and analyzing specific cohorts. General screening is only successful, if the screening tool is frequent and the screening test is highly sensitive and specific. On the other side we want to detect several cardiac diseases at early stages, because we often have good treatment options, i. e. in amyloidosis and Fabry’s diseases.

*Prof. Dr. Andreas Hagendorff (Leipzig):* Considering this problem, which new or conventional echocardiographic parameters should be determined?

*Priv.-Doz. Dr. Daniel Lavall (Leipzig):* End systolic LV elastance and arterial elastance are important parameters to understand myocardial function in different HF types. They allow comprehensive evaluation of LV performance for scientific purposes. Due to the complex calculation of these parameters, strain-based measurements such as strain-work analysis, LV and LA strain may be more helpful for rapid, easy, and reproducible assessment of myocardial function in patients with sign and/or symptoms of HF.

*Prof. Dr. Andreas Hagendorff (Leipzig):* With respect to DD how to analyze LA morphology nowadays?

*Priv.-Doz. Dr. Christoph Sinning (Hamburg):* The conventional approach analyzing LA volumes and the parameter LAVI is the biplane planimetry using 2D echocardiography. Due to the deviation of the longitudinal LA and LV axis the respective atrial volumes are often underestimated by 2D echocardiography. Thus, if possible, we should use 3D volumetry of the cardiac cavities by TTE in our echo labs.

*Prof. Dr. Andreas Hagendorff (Leipzig):* What do you think about the coincidence of AF in patients with “HFpEF” symptoms? Do we need different interpretations of the echocardiographic parameters of LA volumes and DD?

*Priv.-Doz. Dr. Ertunc Altiok (Aachen):* AF is often observed in patients with “HFpEF” symptoms, because increasing LA pressure, impairing LV filling, and stretching LA wall will trigger AF development. However, like the term “HFpEF” AF is mostly a symptom of an underlying disease, which must be characterized by a specific diagnosis. The only exception is lone AF. AF—especially chronic AF—of course, influences LA morphology and function. Thus, LA volume cut offs are different in chronic AF patients in comparison to patients with sinus rhythm.

*Prof. Dr. Andreas Hagendorff (Leipzig):* If we focus on RA and RV analysis, how to improve echocardiographic workflow?

*Dr. Nicolas Merke (Berlin):* There are at least to main important aspects to focus. Firstly, due to the bipyramidal anatomy of the right ventricle we are unable to standardize 2D assessments for RV volume analysis. Thus, we must implement 3D echocardiography for verifiable assessment of RV volumes, Secondly, cardiologists should assess the non-invasive estimation of pulmonary vascular resistance (PVR) in clinical routine to differentiate the causes of pulmonary hypertension. The easiest approach is the formula PVR (cm^−1^) = (10 × Vmax_TR_)/VTI_RVOT_, where VTI_RVOT_ is velocity time integral of flow velocities determined in the right ventricular outflow tract.

*Prof. Dr. Andreas Hagendorff (Leipzig):* Do you think that the structural echocardiographic approach in patients with “HFpEF” symptoms described in the present proposal might substantially improve workflow in clinical routine?

*Prof. Dr. Fabian Knebel (Berlin):* Systematic and structured procedures always have the chance for improvement. The important message of this expert proposal is not to be content to diagnose “HFpEF”. We must focus on the underlying diseases of the “HFpEF” syndrome using all echocardiographic tools. To solve this challenge echocardiography provides more than E/E’. Especially modern tools like deformation imaging and 3D TEE, but also “old” conventional parameters like LV elastance and PVR as well as diastolic stress tests should be integrated into a comprehensive echocardiography.

*Prof. Dr. Andreas Hagendorff (Leipzig):* Carsten, as the coauthor of the HFA-PEFF diagnostic algorithm published 2019 in the European Heart Journal, do you agree with the proposed echocardiographic implementations to analyze patients with “HFpEF” symptoms by TEE?

*Prof. Dr. Carsten Tschöpe (Berlin):* This expert proposal includes a lot of information—perhaps too much for physicians who are not experts in echocardiography. However, the variability of diseases in patients with “HFpEF” favours a more detailed echocardiographic analysis than estimating LVEDP by E/E´ and parameters of DD. As I said in the paper of 2019 “HFpEF” is a syndrome and needs final clarification by a specific diagnosis. Thus, I can agree to prepone the step 4 in our HFA-PEFF algorithm if we start with TTE. The echocardiographic workflow can be more successful in detecting specific diagnoses in patients with “HFpEF” symptoms by a structured approach as introduced in this expert proposal.

*Prof. Dr. Andreas Hagendorff (Leipzig):* To come to an end in our discussion, just a few words to therapeutic options in patients with “HFpEF” symptoms.

*Priv.-Doz. Dr. Sebastian Ewen (Homburg/Saar):* The answer is easy. Specific findings and diagnoses require specific treatment. Thus, prior to treatment the diagnosis is needed. We often treat symptoms, but it is always better to treat the underlying disease than only the symptoms. Thus, if we can address specific cohorts of patients with “HFpEF” symptoms to specific diseases i. e. due to hypertensive heart disease we can analyze therapeutical effects in these patient cohorts better than analyzing a mixture of patients with a variety of underlying diseases with varying prognoses. However, to finalize our discussion, Andreas, what is your final summary to the “HFpEF” topic.

*Prof. Dr. Andreas Hagendorff (Leipzig):* I think the most important message of the expert proposal for the old and young generation of physicians is not to use the term “HFpEF” as a diagnosis anymore. The detection of a specific diagnosis is not only the main target in diagnostics. It also reflects the physician’s competence.
